# Birth–Death Dynamics of Microsatellites: Mechanistic Insights from Orthologous Loci in Felidae

**DOI:** 10.3390/genes16091115

**Published:** 2025-09-19

**Authors:** Wenping Zhang, Mingchun Zhang, Hao Liu

**Affiliations:** 1 Key Laboratory of Research and Conservation of Biological Diversity in Minshan Mountain of National Park of Giant Pandas at Mianyang Normal University of Sichuan Province, College of Life Science, Mianyang Normal University, Mianyang 621000, China; zhang_zoology@163.com; 2 Forest Ecology and Conservation in the Upper Reaches of the Yangtze River Key Laboratory of Sichuan Province, College of Life Science, Mianyang Normal University, Mianyang 621000, China; 3 China Conservation and Research Center for the Giant Panda, Chengdu 610081, China; nbaliker2@163.com

**Keywords:** microsatellite, evolution, Felidae, genome

## Abstract

**Background/Objectives**: The mutational dynamics of microsatellites over deep evolutionary timescales are poorly understood. This study aims to elucidate the life history of trinucleotide microsatellites by tracing orthologous loci across divergent vertebrate lineages and characterizing their mutational pathways. **Methods**: We developed a bioinformatic framework for identifying orthologous microsatellite loci using conserved flanking sequences. This approach was applied to three trinucleotide microsatellites located in exonic, intronic, and intergenic regions, respectively. These loci were amplified and sequenced across 126 individuals representing 64 vertebrate species, whose divergence times range from 6 to 150 million years ago (MYA). **Results**: Flanking sequences proved essential for reliable orthology assignment, while repeat motifs revealed distinct mutational pathways. Microsatellite decay occurs through two primary mechanisms: the complete loss of dominant repeats or their progressive reduction to solitary units (≤1 repeat). This degeneration process is facilitated by cryptic simple sequences (CSS), which act as genomic catalysts promoting birth–death transitions. Large intra-motif deletions were identified as the key mutational event driving contractions and eventual locus degeneration. Furthermore, mutational patterns were highly locus-specific, influenced by genomic context. **Conclusions**: Although the study focused on only three loci, limiting broader generalizations, our findings provide mechanistic insights into microsatellite evolution. These results establish a foundation for modeling complex microsatellite life histories and highlight the role of CSS in facilitating evolutionary turnover.

## 1. Introduction

Microsatellites, which consist of short tandem repeats (STRs) of 2–6 nucleotides, are widespread components of eukaryotic genomes, though their structure, length, and abundance vary across species and genomic regions [1,2]. They are notably enriched in centromeric and telomeric regions and are present in both coding and non-coding gene sequences [3,4]. Within coding regions, functional constraints exert stronger selective pressures on microsatellites to maintain protein integrity [5].

Growing evidence highlights the functional importance of microsatellites. Repeat length polymorphisms influence a wide range of traits, such as limb and skull morphology in dogs [6], behavioral phenotypes in voles [7], and gene expression through alterations in chromatin structure and epigenetic modifications in humans [8,9,10]. Pathogenic repeat expansions, defined as significant deviations from the population mean repeat number, are associated with over 40 human neurological, neurodegenerative, and neuromuscular disorders [11,12,13,14,15,16].

Due to their high polymorphism and ease of genotyping, microsatellites also serve as invaluable genetic markers in population, forensic, and conservation genetics [17]. Their applications range from individual and pedigree identification to reconstructing population histories [18], and they can even act as molecular clocks for coalescent events spanning millions of years [19]. Many of these applications rely on evolutionary models that simulate mutational processes at microsatellite loci [20].

Studies on microsatellite evolution mechanisms propose a life cycle involving birth, expansion, contraction, death, and potential resurrection. Suggested mechanisms include slipped-strand mispairing, which initiates expansion beyond a critical threshold (“birth”) [21], dual pathways involving expansion of existing repeats or conversion of cryptic simple sequences [22], mutation biases associated with recombination hotspots [23], and length-dependent increases in slippage probability that drive expansion [17,24,25,26]. In contrast, point mutations that interrupt repeats can hinder slippage and lead to “death” [27], often involving large deletions that leave little homology to the original repeat [18,24,28].

A key unresolved issue is the exact minimal repeat number (“threshold”) required for microsatellite birth and death dynamics [24,29]. Although whole-genome sequencing has provided insights into mutation processes e.g., [24,27,30], available data remain inadequate for detailed characterization and threshold determination. Previous studies of “death” have often focused on partial motif interruption via point mutations, rather than complete loss or failure to reach a true threshold, e.g., [24,31]. Therefore, tracking the dynamics of orthologous microsatellite loci across broad evolutionary clades is essential.

Felid species present an ideal model for such investigations. Despite extensive microsatellite research in various organisms, felids have been relatively understudied. They share a common ancestor within the last 10 million years and exhibit a highly conserved genome structure [32]. Their well-resolved molecular phylogeny [32,33,34] makes them particularly suitable for studying gradual change in microsatellite mutation over extended evolutionary timescales.

In this study, we focus on trinucleotide microsatellites (tri-STRs). Tri-STRs are relatively common in coding regions because insertions or deletions of a single repeat unit add or remove one amino acid without disrupting the reading frame. This feature enables comparative analysis of evolutionary mechanisms between coding and non-coding regions. Accordingly, we used sequence analysis to examine the mutation patterns at three orthologous tri-STR loci—located in an exon, an intron, and a non-coding region—across *Felidae* species.

## 2. Materials and Methods

### 2.1. DNA Samples

Genomic DNA was extracted from whole blood samples collected from 66 adult individuals (Table 1). Approximately 5 ml whole blood was obtained from each individual during routine physical examinations conducted by veterinarian practitioners. For small felids, physical restraint using net bags was employed during blood collection; for large felids, anesthesia was administered according to body weight prior to sampling. The 66 individuals represented four major felid lineages (Panthera, Bay cat, Leopard, and Domestic cat lineages), as defined by Johnson et al. (2006) [33]. DNA extraction was performed using a standard proteinase K digestion and phenol–chloroform protocol (Sambrook et al., 1989) [35].

### 2.2. Microsatellite Amplification and Sequencing

#### 2.2.1. Locus Selection and Amplification

Two microsatellite loci were amplified and sequenced:

Ptia5: This is a non-coding locus yielding PCR products approximately 250 bp in length. Amplification followed the protocol of Zhang et al. (2006) [36]. PCR products were ligated into the pGEM-T Easy Vector (Promega, Madison, WI, USA) and transformed into Escherichia coli DH5α competent cells. To minimize artifacts, 30 positive clones per sample were selected for Sanger sequencing.

Ptia2-intron: This is located within an intron of *NCBP3*, producing PCR products approximately 150 bp in length. The primers used were as follows: Ptia2-F (5′-TCCGAAGTCTGTCCTGTT-3′) and Ptia2-R (5′-AGTTGGGAATGCTGCTC-3′). Based on evidence that NGS read counts accurately reflect mixture proportions [37], this locus was prepared for Roche 454 GS-FLX sequencing. Each sample was tagged with a unique 4-bp barcode.

#### 2.2.2. PCR Conditions

PCR reactions for both loci were performed using 2× High-Fidelity PCR Master Mix (MedChemExpress, Shanghai, China) in a 20 μl total volume containing: 15 pmol each of forward and reverse primer, 200 μM each dNTP, 3.5 μl of 1 μg/μl bovine serum albumin (BSA), approximately 50 ng template DNA, 1 U DNA polymerase (MBI Fermentas, Burlington, ON, USA), and 0.6 μl of 25 mM MgCl_2_. Amplification was performed on a GeneAmp^®^ 9700 thermal cycler (Applied Biosystems, Waltham, MA, USA) under the following conditions with a touchdown method: initial denaturation at 95 °C for 7 min; 40 cycles of denaturation at 94 °C for 30 s, annealing at 56–47 °C (2 cycles per temperature) and 50 °C (20 cycles) for 45 s, and extension at 72 °C for 55 s; with a final extension at 72 °C for 30 min.

#### 2.2.3. NGS Library Preparation and Sequencing (Ptia2-Intron Only)

PCR products for Ptia2-intron were quantified, normalized to equimolar concentrations, and pooled. Pool integrity was verified via 2% agarose gel electrophoresis, and the pool was purified using the Qiagen Gel Extraction Kit (Qiagen, Hilden, Germany). Sequencing libraries were constructed from the purified pool and sequenced on a Roche 454 GS-FLX platform following Malausa et al. (2011) [38]. A minimum of approximately 60 high-quality sequence reads per sample was targeted to ensure sufficient coverage and cost-effectiveness, based on bead loading estimates.

#### 2.2.4. Sequence Processing and Genotyping

After demultiplexing sequences from both loci (Sanger reads for Ptia5 and 454 reads for Ptia2-intron), microsatellite sequences and genotypes were identified for each sample and locus using a bioinformatics pipeline [39] integrated with the FDSTools analysis toolkit [40].

### 2.3. Orthologous Locus Identification and Sequence Acquisition

#### 2.3.1. Exonic Locus (Ptia2-Exon)

An exon (NCBP3-201 ENSPTIE00000059046, 298 bp) containing a GAG repeat motif within the gene *NCBP3* was identified and retrieved from the tiger (*Panthera tigris*) genome assembly. This microsatellite locus was designated as Ptia2-exon.

#### 2.3.2. Ortholog Detection Across Vertebrates

BLASTN searches (using the “somewhat similar sequences” option) were conducted against reference genomes of 62 vertebrate species (Table S1) to identify orthologs of all three loci (Ptia5, Ptia2-intron, Ptia2-exon) across a broad evolutionary scale. These species selected represent major vertebrate classes and most carnivore families. High-quality genomic resources were used as the reference database.

#### 2.3.3. Sequence Retrieval and Validation

Significant alignments were defined as those with 100% query coverage and >80% nucleotide identity. Putative orthologs were manually inspected to confirm homology to the target loci and then downloaded. The genomic context (e.g., exon, intron, intergenic) of each identified locus was determined based on the BLAST (2.17.0) alignment with the lowest E-value. Loci lacking significant BLAST hits for both flanking regions were excluded.

### 2.4. Sequence Alignment and Polymorphism Analysis

Multiple sequence alignments for haplotypes of the three loci were performed using BioEdit version 7.0.2 [41]. Alignments within microsatellite repeat and flanking regions were manually refined to accommodate point mutations and small indels. Within the microsatellite region, only repeat units with identical motifs across multiple species or individuals were aligned; adjacent sequences were classified as flanking regions. For each locus, polymorphism was characterized by quantifying (1) variation in repeat unit number (allele size), (2) single-nucleotide polymorphisms (SNPs), and (3) insertion/deletion (indel) events within both the repeat motif and the flanking regions.

### 2.5. Variation in Repeat Unit Motifs

To examine variation in repeat unit motifs across Felidae species at the three loci, microsatellite allele repeats were mapped onto a fully resolved phylogenetic tree of Felidae [33,34], with the giant panda used as an outgroup. Phylogenetic relationships among tiger subspecies were based on Sun et al. (2023) [42]. Note that branch lengths are not proportional to evolutionary distance. Allele repeats from species in Table 1 were combined with unit repeats from reference genomes listed in Table S1. Repeat unit motifs and lineage classifications were visualized using the toolkit package v.1.1.9 [43] in R and the online tool iTOL v.6 [44].

### 2.6. Statistical Characterization of Microsatellite Mutation

#### 2.6.1. Lineage-Level Mutation Patterns

**Figure 1:**
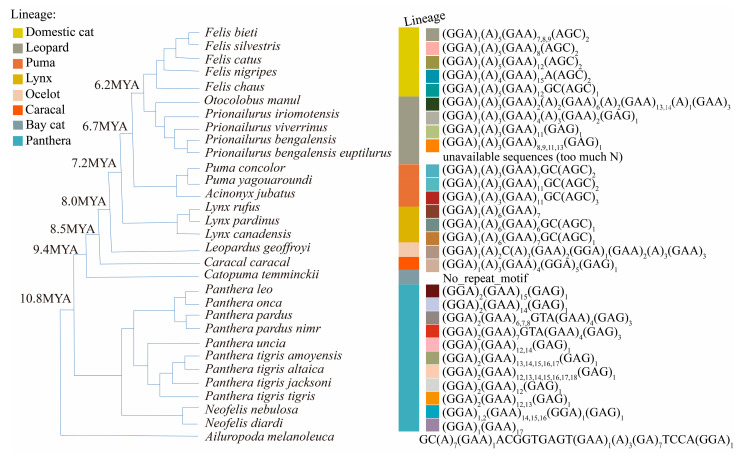
Variation in repeat unit motifs at locus Ptia5 across Felidae species. The outermost bar uses color-coded segments to represent distinct repeat unit motifs, while the adjacent color blocks indicate lineage taxonomic classifications [33,34].

**Figure 2:**
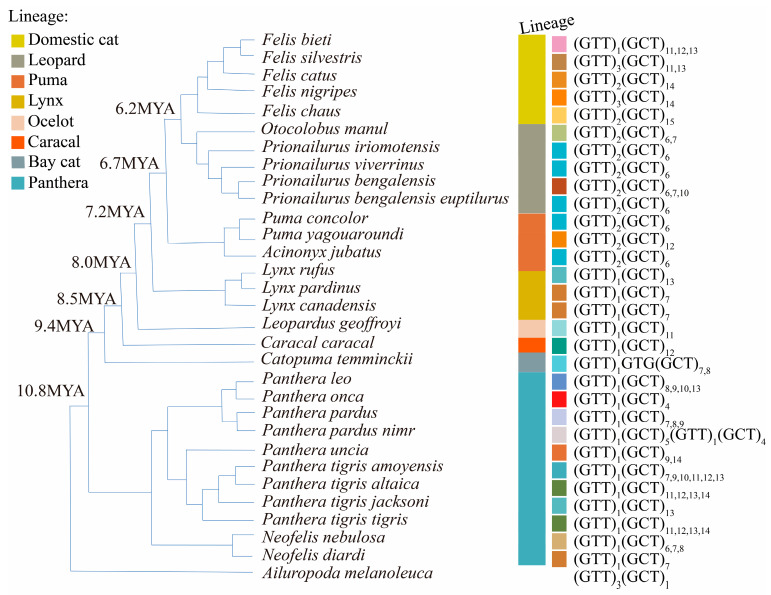
Variation in repeat unit motifs at the Ptia2-intron locus among Felidae species. Color-coded segments in the outer ring represent distinct repeat units, while adjacent colored blocks indicate lineage taxonomic classifications [33,34].

**Figure 3:**
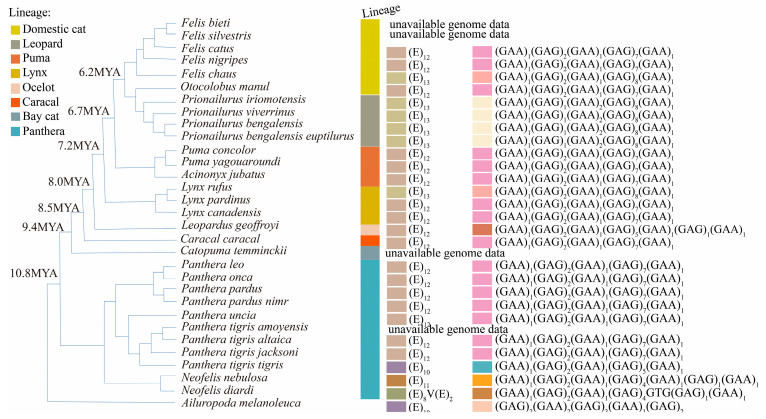
Variation in repeat unit motifs at the Ptia2-exon locus across Felidae species. Color-coded segments in the outermost bar represent nucleotide repeat sequences, with corresponding amino acid repeats shown in adjacent tracks. Lineage taxonomic classifications [33,34] are indicated by colored blocks. All allele repeats were sourced from reference genomes available in NCBI; further details are provided in Table S1.

**Figure 4:**
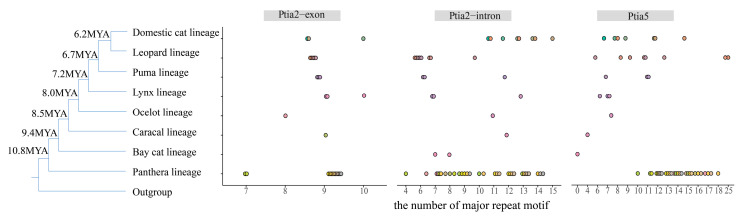
Varieties of major repeat motifs in microsatellite alleles across Felidae lineages at three loci. Phylogenetic relationships and estimated node divergence dates are from Johnson et al. (2006) [33]. Branch lengths are not proportional to evolutionary distance. Each dot represents one allele. Major repeat motifs per locus are shown: Ptia2-exon for (GAG)_n_, Ptia2-intron for (GCT)_n_, and Ptia5 for (GAA)_n_.

Modern felid species comprise eight distinct lineages [33,34] (Table S1, Figure 1, Figure 2, Figure 3 and Figure 4). The consensus repeat number of the major motif for each microsatellite allele across the three loci was mapped onto the established felid phylogenetic tree [33] to illustrate mutation patterns following lineage radiation (Figure 4).

#### 2.6.2. Mutation Model Testing

The mutational behavior of each microsatellite locus was evaluated by comparing the Stepwise Mutation Model and the Multi-Step Mutation Model using the program MISAT [45]. Analyses were conducted under default settings using a maximum likelihood approach.

#### 2.6.3. Statistical Analyses

All statistical analyses were performed using SPSS Statistics for Windows (Version 13.0, IBM Corp., Armonk, NY, USA) or Microsoft Excel. Analysis of variance (ANOVA) was used to test for significant differences in repeat number among the eight felid lineages.

Moreover, Bayes Factor tests were applied to evaluate the support for threshold effects in microsatellite decay (e.g., ≤1 repeat unit as a “death” state).

## 3. Results

### 3.1. Mutation in Non-Coding Microsatellite Locus Ptia5

We obtained 1980 sequences for the Ptia5 locus from the 66 individuals via Sanger sequencing (Table 1). Sequences lacking microsatellite motifs were excluded. The remaining sequences were genotyped, and allele were identified using the bioinformatics pipeline described by Lepais et al. (2020) [39]. This process yielded 1972 sequences containing Ptia5 microsatellites, which represented 34 alleles across the 66 individuals. In addition, 32 orthologous Ptia5 sequences were obtained from the NCBI database for species with publicly available genomic resources (Table S1). For the Amur tiger (ALT) and South China tiger (SCT) populations, which each comprise more than 10 individuals (Table 1), locus Ptia5 revealed seven alleles in ALT and five in SCT. Both populations showed high heterozygosity, and low genetic differentiation was observed between SCT and ALT for this locus (Table S2).

Ptia5 is a compound microsatellite locus characterized by a major (GAA)_n_ repeat unit in all examined *Felidae* alleles, except in *C. temminckii*, which showed a large deletion within the repeat motif (Figure 1) and a consistent 40 bp deletion in the flanking sequence across all individuals (Figure S1). Interruptions in the major repeat unit were detected in *Leopardus geoffroyi*, *P. pardus*, *P. pardus nimr*, *P. iriomotensis*, and *O. manul* (Figure 1). The repeat motif displayed lineage-specific characteristics within *Felidae*: polyA stretches were present in six of the eight lineages (absent in the Panthera and Bay cat lineages), ranging from 2 As (Ocelot lineage) to 6 As (Lynx lineage). Additionally, the repeat motif began with one GGA in six lineages (excluding Bay cat and Panthera), while the Panthera lineage contained either one or two GGAs (Figure 1, Table S1). Novel repeat motifs emerged at the end of the repeat region in certain lineages: (GAG)_n_ in the Panthera, Caracal, and Leopard lineages, and (AGC)_n_ in the Lynx, Puma, and Domestic cat lineages (Figure 1, Table S1).

Orthologous Ptia5 sequences were also identified in *Hyaenidae*, *Ursidae*, and *Viverridae* (Table S1). In the three *Hyaenidae* species, the major (GAA)_n_ repeat—typical of *Felidae*—was substantially reduced (two repeats in *Crocuta crocuta*, one in *Proteles cristata cristata*, zero in *Hyaena hyaena*) and interrupted by polyG preceding and polyA within the original repeat region. No trinucleotide repeat motifs longer than two units were detected in these *Hyaenidae* sequences (Table S1). In the giant panda (*Ailuropoda melanoleuca*, *Ursidae*), the corresponding region was replaced by a (GA)_7_ motif (Figure 1, Table S1). In *Viverridae* (*Paguma larvata* and *Paradoxurus hermaphroditus*), the homologous repeat region consisted of cryptic simple sequences (GCGTGGGAGGGGCAGAGAA and GCGTGAGAGGGGCAGAGAA, respectively) (Table S1). No significant Ptia5 orthologies were identified outside *Carnivora*. Across all species analyzed, the longest uninterrupted nucleotide repeat spanned 18 units (Table S1).

### 3.2. Mutation in Intron Microsatellite Locus Ptia2-Intron

Sequencing the Ptia2-intron locus in 66 individuals (Table 1) using Roche 454 GS-FLX produced 1764 sequences. After removing short sequences and those lacking microsatellite motifs, 1578 sequences (mean 24 ± 10 per sample) remained for genotyping, which identified 38 alleles. Additionally, 35 orthologous Ptia2-intron sequences were retrieved from NCBI (Table S1). Similarly, for Ptia2-intron, four alleles were identified in ALT and six in SCT. This locus also exhibited high heterozygosity in both populations, along with low genetic differentiation between them (Table S2).

Ptia2-intron is a compound microsatellite containing a major (GCT)_n_ repeat unit in all *Felidae* alleles. Interruptions within this major repeat were observed in *C. temminckii* and *P. pardus nimr*. The copy number of a minor (GTT)_n_ motif exhibited lineage-specific variation within *Felidae*: the Domestic cat lineage carried one, two, or three repeats; the Leopard and Puma lineages consistently had two repeats; and the Lynx, Ocelot, Caracal, Bay cat, and Panthera lineages each contained only one repeat (Table S1).

Orthologous Ptia2-intron sequences were also identified in *Hyaenidae*, *Ursidae*, *Viverridae*, *Mustelidae*, and *Procyonidae* (Table S1). Notably, *Mustelidae* and *Procyonidae* lacked significant Ptia5 homologs. In non-*Felidae* species, the copy number of the (GCT)_n_ motif decreased from 4 to 0 as the divergence in the flanking sequences of Ptia2-intron increased (Table S1; Figure S1). The major (GCT)_n_ motif was absent in *Neovison vison* (*Mustelidae*). The copy number of the minor (GTT)_n_ motif was similar between Felidae and non-Felidae carnivores (Figure 2; Table S1). No significant Ptia2-intron orthologs were detected outside *Carnivora*. The longest uninterrupted nucleotide repeats at this locus consisted of 15 units (Table S1).

Within each species, only a single indel is required to transition from one allele to a progressively longer or shorter allele at both the Ptia5 and Ptia2-intron loci, suggesting that stepwise mutation occurred within the tandem repeat regions of each species.

### 3.3. Mutation in Exon Microsatellite Locus Ptia2-Exon

We obtained 60 orthologous Ptia2-exon sequences from NCBI (Table S1), representing species spanning divergence times of hundreds of millions of years. A highly variable region was identified within the Ptia2-exon flanking sequence (Figure S1). In contrast to other loci, the Ptia2-exon flanking sequences were highly conserved within *Felidae*; sequences from species across different genera—including *F. chaus*, *F. nigripes*, *Acinonyx jubatus*, *P. concolor*, *P. yagouaroundi*, *L. canadensis*, *L. pardinus*, *L. rufus*, and *Caracal caracal*—were identical (Figure S1).

Ptia2-exon is a compound microsatellite encoding glutamic acid (Glu, E), consisting of (GAA)_n_ and (GAG)_n_ repeats. The (GAG)_n_ repeat, which occurred in higher copy numbers, constituted the major repeat unit in all *Felidae* alleles. This resulted in polyglutamic acid (polyE) tracts ranging from 10 to 13 residues in *Felidae* species, except in *N. diardi*, where the motif was interrupted by GTG, substituting valine (Val, V) for glutamic acid (Figure 3). The amino acid and nucleotide sequences of the repeat motif were identical within *Prionailurus* species but varied among species of other genera (e.g., *Panthera*, *Felis*, *Lynx*) (Figure 3; Table S1). The repeat tracts in *Felidae* consistently began and ended with (GAA)_n_ (Figure 3; Table S1). The predominant repeat sequence, (GAA)_1_(GAG)_2_(GAA)_1_(GAG)_7_(GAA)_1_, was observed in 15 *Felidae* species. A secondary major sequence, (GAA)_1_(GAG)_1_(GAA)_2_(GAG)_8_(GAA)_1_, was found in six species (Figure 3; Table S1).

In non-Felidae species, the (GAA)_n_ and (GAG)_n_ motifs were interrupted by GAT and/or GAC in *Ambystoma mexicanum*, *Scyliorhinus canicula*, *Leucoraja erinacea*, and *Phascolarctos cinereus*. These substitutions replaced glutamic acid with aspartic acid (Asp, D), resulting in novel (D)_n_ amino acid repeats and nucleotide motifs, such as (GAT)_2_ and (GACGAG)_2_ (Table S1). The nucleotide repeat tracts in these species began and ended with either (GAA)_n_ or (GAG)_n_, contrasting with the consistent (GAA)_n_ termini observed in *Felidae*. The number of amino acid residues in the repeat region ranged from 8 to 17, with *Hominidae* exhibiting the longest tract (Table S1). The longest stretch of uninterrupted (GAA)_n_ or (GAG)_n_ repeats was only two units, observed in *Neovison vison*, *Potos flavus*, *Talpa europaea*, *Ambystoma mexicanum*, *Oceanodroma leucorhoa*, and *Scyliorhinus canicula*. However, the longest uninterrupted amino acid repeat (polyD or other) reached 12 units in these species (Table S1). Overall, the longest uninterrupted amino acid repeat observed was 17 units, while the longest uninterrupted nucleotide repeat was 8 units (Table S1). These findings indicate that the amino acid sequence (polyE) encoded by Ptia2-exon is highly conserved and has been maintained over hundreds of millions of years of divergence (Table S1), while the underlying nucleotide sequences show higher mutation rates within the repeat region.

Analysis of all alleles across the three loci using CENSOR [46] revealed no proximity to transposable elements, specifically non-LTR retrotransposon CR1.

### 3.4. Variation in Repeat Region Length Across Felidae Lineages

Modern felid radiation originated in Asia approximately 10.8 MYA, with the divergence of the Panthera lineage from the common ancestor of other felids [33,34]. Subsequent divergences occurred in the following order: Bay cat lineage, Caracal lineage, Ocelot lineage, Lynx lineage, Puma lineage, Leopard lineage, and most recently, the Domestic cat lineage [33,34] (Figure 4).

For Ptia5, Pallas’s cat (*O. manul*) exhibited the longest allele size, whereas the Asiatic golden cat (*C. temminckii*) had the shortest among *Felidae* species (Figure 1). Among lineages with data from ≥3 species (Panthera, Leopard, Domestic cat), significant differences in Ptia5 repeat size ranges were observed only between the Panthera and Domestic cat lineages (*p* < 0.05, ANOVA). A pattern of contraction from the Panthera lineage to the Bay cat lineage, followed by expansion toward the remaining lineages, was evident (Figure 4). This expansion and contraction were primarily driven by changes in the number of (GAA)_n_ repeat, despite some species-specific variations (Figure 1 and Figure 4). Repeat size differences between the Leopard and Domestic cat lineages were not significant (*p* = 0.4866, ANOVA), indicating no net expansion or contraction between them.

For Ptia2-intron, the (GCT)_n_ repeat size was shortest in the jaguar (*P. onca*) and longest in the jungle cat (*F. chaus*) (Figure 2). Allele repeat sizes and ranges showed extensive overlap between the Panthera lineage and other Felidae lineages (Figure 2 and Figure 4), suggesting no significant expansion or contraction from Panthera to other lineages (Figure 4). Although data were limited for direct comparison between the Bay cat and Caracal lineages, a visible expansion from the Bay cat to the Caracal lineage was observed (Figure 4). Moreover, a highly significant difference in repeat lengths was detected between the Leopard and Domestic cat lineages (*p* < 0.001, ANOVA), indicating significant expansion from the former to the latter for Ptia2-intron (Figure 4).

For Ptia2-exon, allele repeat sizes and ranges overlapped considerably across Felidae lineages. The major (GAG)_n_ motif consisted predominantly of 9 (23 out of 28 species), while two species had 7 repeats, two had 10 repeats, and one species had 8 repeats (Figure 3 and Figure 4). Differences in repeat sizes between lineages were not statistically significant (*p* > 0.05 for all pairwise comparisons, ANOVA), indicating that Ptia2-exon did not undergo significant expansion or contraction across felid lineages.

### 3.5. Statistical Tests of the “Threshold” for Births/Deaths of Microsatellites

To quantitatively assess the hypothesis that microsatellite loci undergo evolutionary “death” below a critical repeat number threshold, we performed a Bayesian model comparison between a threshold model and a continuous model. The threshold model specified a distinct, abrupt change in the probability of locus death at a repeat number of ≤1, whereas the continuous model described a gradual, linear relationship between repeat number and death probability using a logistic function.

Bayesian model comparison, evaluated using Bayes Factors (BFs), provided very strong evidence in favor of the threshold model. The analysis yielded a Bayes Factor (BFThreshold/Continuous) of 85.2 (2lnBF = 8.9) (Table 2), which strongly supports the model containing a discrete threshold over the continuous alternative.

Parameter estimates from the threshold model were highly consistent with the proposed biological mechanism. The posterior mean probability of death for loci at or below the threshold (repeat number ≤ 1) was estimated at p_high = 0.92 (95% Credible Interval [CI]: 0.83–0.97). In stark contrast, the probability of death for loci above this threshold was markedly low, with a posterior mean of p_low = 0.07 (95% CI: 0.02–0.14). The sharp, step-like difference in these probabilities is not adequately captured by the smooth function of the continuous logistic model, which failed to identify a specific critical point of failure.

## 4. Discussion

In this study, we identified orthologous microsatellite loci across representative vertebrate species covering major Carnivore families through systematic BLASTN searches and amplicon analysis. This approach enabled precise mapping of orthologous sequences to elucidate microsatellite mutation dynamics across species. Our findings indicate that flanking sequences are critical for determining orthology, while repeat motifs offer key insights into microsatellite life cycles—a methodological framework underrepresented in prior studies, e.g., [47]. We identified polymerase slippage-induced stepwise changes in copy number as the primary mutational mechanism within species (Figure 1 and Figure 2), consistent with established models [20,25,48]. Notably, microsatellites exhibited significantly higher per-locus mutation rates compared to single nucleotide variants (SNVs) in flanking regions (Figure 1, Figure 2 and Figure 3 and Figure S1), corroborating earlier reports [25,49]. Methodologically, next-generation sequencing (NGS) proved more cost-effective than Sanger sequencing while maintaining comparable accuracy.

### 4.1. Constraints on Maximum Repeat Numbers

The observed upper limit of microsatellite expansion (≤50 repeats) aligns with theoretical predictions [50,51,52]. Specifically, maximum trinucleotide repeats were 25 for Ptia5 (Pallas’s cat), 17 for Ptia2-intron (jungle cat), and 17 for Ptia2-exon (chimpanzee). Constraints for perfect repeats were further refined: (GAA)_n_ ≤ 18, (GCT)_n_ ≤ 15, and (GAG)_n_ ≤ 8. While point mutations typically fragment long alleles [53]—as observed in Ptia2-exon and Ptia5—the exceptional stability of Ptia2-intron (Figure 2) suggests locus-specific selective pressures. This divergence implies that intrinsic deleterious effects may drive microsatellite size reduction through negative selection.

### 4.2. The “Threshold” for Births/Deaths of Microsatellites

The concept of a minimum repeat threshold is fundamental to understanding microsatellite birth and death dynamics, yet inconsistent definitions across studies have led to divergent conclusions [24,29]. Thresholds for mononucleotide microsatellites range from 7–9 repeat units, while di- to tetra-nucleotide microsatellites exhibit thresholds of 4–8 units [24,54,55]. Alternative approaches define thresholds by absolute nucleotide length (e.g., 12 bp) regardless of motif size [56]. Although the importance of the threshold is widely acknowledged, its precise value remains contentious [53,57,58]. Our findings suggest that this threshold should not exceed one repeat unit, as elaborated below.

Large-scale deletions represent a primary mechanism of microsatellite death. Such deletions may eliminate repeat motifs, as observed in the Bay cat lineage following its divergence from Panthera. Here, Ptia5 underwent death in the Bay cat lineage via deletion of its core repeat motif (Figure 1 and Figure 4) and a 40 bp flanking sequence (Figure S1), with subsequent resurrection evidenced by homologous motifs in other felid lineages. Deletions may alternatively leave residual motifs, exemplified by trinucleotide repeats in *Hyaenidae* (excluding (G)n/(A)n), where the dominant felid (GAA)_n_ motif was either absent or reduced to a single repeat in *Hyaena hyaena* and *Proteles cristata*. Identical Ptia5 sequences from two *H. hyaena* genomes (GCA_003009895.1, GCA_004023945.1) confirm locus death. Similarly, deletion of the (GCT)_n_ motif in Ptia2-intron eliminated all GCT repeats in *N. vison* and reduced them to one unit in *P. flavus* and *A. melanoleuca*. Uniform sequences across three *N. vison* genomes (GCA_020171115.1, GCA_964106205.1, GCA_900108605.1) further validate locus death.

A second death mechanism involves mutation accumulation that disrupts tandem repeats below functional thresholds, generating highly CSS with nucleotide composition biases as defined by Tautz et al. (1986) [59]. In *Viverridae*, the Ptia5 repeat motif was replaced by 19 bp CSS variants ([GCGTGAGAGGGGCAGAGAA] in *Paradoxurus hermaphroditus*; [GCGTGGGAGGGGCAGAGAA] in *Paguma larvata*), each retaining just one GAA repeat. This CSS exhibits high cryptic simplicity and resides within conserved flanking sequences (9 SNPs/234 bp), indicating the death of Ptia5 in *Viverridae*. We propose that these CSS regions act as “genomic seeds,” prone to replication slippage and facilitating the de novo generation of new microsatellites [18,22,60].

Repeat array interruption represents a third pathway, particularly in coding regions, as demonstrated by Ptia2-exon. In *Ambystoma mexicanum*, with two genomes in NCBI (GCA_002915635.3, GCA_040938575.1), the repeat of the two sequences of Ptia2-exon degenerated to (GAG)_1_(GAA)_1_(GAG)_1_(GAA)_1_(GAG)_1_(GAT)_2_GAC(GAG)_1_, reducing dominant felid units (GAG/GAA) to single repeats. Synonymous codon usage (GAG/GAA for glutamic acid) may minimize slipped-strand mutations while stabilizing protein domains essential for protein–protein interactions and complex assembly [61,62].

Notably, *A. melanoleuca* exhibited reduction of the Ptia5 trinucleotide motif to one repeat alongside emergence of a novel (GA)_7_ dinucleotide motif. Distinct (GA)_n_ alleles in two genomes (GCA_002007445.3, GCA_029963865.1) suggest simultaneous locus death and rebirth, echoing findings by Buschiazzo and Gemmell (2006) [18].

Collectively, these results reveal diverse microsatellite death pathways across taxa, supporting the model of a flexible life cycle proposed by Shortt et al. (2020) [29]. Crucially, point mutations alone may not constitute death; rather, loss of major repeat units or reduction to ≤1 repeat defines death [28,58]. This aligns with Taylor et al. (1999)’s [28] framework, in which death follows either large deletions or mutation-driven degradation below threshold lengths, and Leclercq et al. (2010)’s [58] demonstration of persistent slippage in minimal repeats. Although mutation dynamics vary by motif [53,63], our ≤1 threshold finding remains robust despite analysis of only three loci. This is supported by Bayesian analysis, which provides robust statistical evidence for the existence of a discrete threshold at a repeat number of one, beyond which microsatellite loci have a very high probability of entering an evolutionary “death” state characterized by an inability to expand (Table 2).

### 4.3. Directional Evolutionary Trajectories

Our analyses of motif replacement among orthologous felid microsatellites reveal that mutational dynamics differ significantly not only among microsatellites of different motif lengths but also among those with distinct motif compositions. Following the radiation of modern felids, Ptia5 underwent progressive contraction, leading to death in the Bay cat lineage after its divergence from Panthera, followed by resurrection in other lineages. In contrast, Ptia2-intron exhibited expansion exclusively along the Leopard-to-Domestic cat lineage trajectory, while Ptia2-exon showed no detectable expansion, contraction, or extinction events across the surveyed lineages (Figure 4). These observations align with prior studies documenting locus-specific mutational heterogeneity in microsatellites [17,18,64].

### 4.4. Factors Influencing Mutational Dynamics

Multiple factors modulate microsatellite evolution, including local mutation/repair rates, motif size, repeat number, and genomic position [65,66]. Our analyses revealed distinct conservation patterns corresponding to genomic context: the noncoding locus Ptia5 persisted in lineages diverging about 10–20 MYA, the intronic Ptia2-intron in lineages diverging about 20–30 MYA, and the exonic Ptia2-exon in lineages diverging about 150–200 MYA (Figure 1, Figure 2 and Figure 3, Table S1). These findings align with those of Wissler et al. (2012) [67], who demonstrated slower evolutionary rates for exonic microsatellites both within and across species, exemplified by the conserved mammalian CGG repeat in the *FMR1* gene [68,69].

As expected, Ptia2-exon, located in an exon of *NCBP3*, exhibited the lowest diversity among the studied loci (Figure 1, Figure 2 and Figure 3, Table S1). This reflects fundamental constraints on coding-region microsatellites, where protein functionality imposes selective pressures that limit variability. In contrast, noncoding microsatellites evolve under distinct selective regimes independent of protein conservation, instead reflecting locus-specific features and regulatory influences [15,47,70].

These results also indicate that the progression through the birth–death cycle is strongly modulated by the genomic environment, explaining why different loci have vastly different evolutionary trajectories and longevities.

### 4.5. Study Limitations and Strengths

This study establishes a comprehensive phylogenetic framework to investigate microsatellite evolution over deep evolutionary timescales (up to 150 million years). By analyzing orthologous loci across 64 species from diverse lineages, we reveal consistent patterns of microsatellite birth, expansion, contraction, and decay, supporting the hypothesis that repetitive DNA evolves through life-cycle-like dynamics. The use of conserved flanking sequences to infer orthology ensured high confidence in locus identification and cross-species comparisons. Moreover, the identification of conserved sequence structures (CSS) as potential catalysts of microsatellite turnover provides a novel mechanistic perspective on the evolutionary dynamics of repetitive genomic regions.

Several limitations must be considered when interpreting these findings. First, the limited sample size per species (ranging from 1 to 23 individuals) constrained accurate estimation of mutation rates and intra-species polymorphism. Second, although the three loci examined represent distinct genomic contexts (exonic, intronic, and intergenic), the small number of loci limits broader generalizations about genome-wide microsatellite behavior. Finally, as a comparative study, our conclusions rely on phylogenetic inference rather than experimental validation of mutational mechanisms. Functional assays or long-read sequencing are needed to explicitly test the proposed models of mutation and selection and to determine whether the observed patterns are stochastic or indicative of a structured life cycle.

Despite these limitations, this work provides a foundation for future studies that integrate phylogenetic reconstruction with population genetic and functional analyses to elucidate the complex life history of microsatellites. Our findings suggest that progression through the birth–death cycle is strongly influenced by genomic context, explaining the divergent evolutionary trajectories and longevities observed across loci. Future research with high-depth population sampling would benefit from (1) phylogenetic comparative methods to correlate repeat stability with lineage-specific mutation rates; (2) likelihood-based models of microsatellite evolution incorporating both expansion and contraction processes; and (3) Bayesian approaches to estimate the timing of major contraction and expansion events.

## 5. Conclusions

This study established a methodological framework for identifying orthologous microsatellite loci across divergent vertebrate species, facilitating systematic analysis of microsatellite mutation dynamics. Our results show that microsatellite length follows cyclical patterns of expansion and contraction over evolutionary timescales, with distinct mutational dynamics that vary by motif length and nucleotide composition. The directional evolution of repeat number further exhibits locus-specific dependencies, underscoring the influence of genomic context. Importantly, we identify cryptic simple sequences as key elements in microsatellite turnover and reveal large-scale deletions as a major mechanism driving locus contraction and death. Microsatellite death occurs when major repeat units are reduced to ≤1 copy—a novel threshold criterion supported by our empirical data. Although the current methodology was validated across three representative loci and further optimization is needed for broader taxonomic application, these findings significantly advance theoretical models of repetitive DNA evolution. The framework provides essential parameters for understanding mutational processes in microsatellites and lays a foundation for future development of automated systems for detecting orthologous microsatellite. Given that this study was limited to three loci, future work should aim to identify multiple orthologous microsatellites at the genome scale across divergent lineages.

## Figures and Tables

**Table 1:** Origins of samples of Felidae used for Ptia5 and Ptia2-intron in this study.

Birth Status	Scientific Name	Common Name	Individuals’ Numbers
Wild	*Felis bieti*	Chinese desert cat	4
Wild	*Felis silvestris*	European wild cat	1
Wild	*Prionailurus bengalensis*	Asian leopard cat	5
Wild	*Catopuma temminckii*	Asiatic golden cat	3
Wild	*Neofelis nebulosa*	Clouded leopard	3
Wild	*Panthera uncia*	Snow leopard	3
Captive and Wild	*Panthera pardus*	Leopard	6
Captive	*Panthera tigris altaica*	Amur tiger	12
Captive	*Panthera tigris tigris*	Bengal tiger	2
Captive	*Panthera tigris amoyensis*	South China tiger	23
Captive	*Panthera leo*	Lion	2
Wild	*Otocolobus manul*	Pallas’ cat	2

**Table 2:** Results of Bayesian model comparison.

Comparison	BF	Log(BF)	2 × Log(BF)	Evidence Strength
Threshold vs. continuous	85.2	4.45	8.90	Strong

## Data Availability

The original contributions presented in this study are included in the article/Supplementary Material. Further inquiries can be directed to the corresponding author.

## Supplementary Materials

The following supporting information can be downloaded at https://www.mdpi.com/article/10.3390/genes16091115/s1, Table S1: The sample information with genomes in this study; Table S2: Summary of genetic diversity statistics between SCT and ALT, including observed (H(O)) and expected (H(E)) heterozygosity and F- statistics; Figure S1: Annotated alignments of microsatellite loci. (a) Ptia5: 5′-flanking region (1–204), repeat motif (204–205, details for Figure 1), 3′-flanking region (205–234). (b) Ptia2-intron: 5′-flanking region (1–80), repeat motif (80–81, details for Figure 2), 3′-flanking region (81–124). (c) Ptia2-exon: 5′-flanking region (1–30), repeat motif (31–91), 3′-flanking region (92–345). Gap symbols: “-“ = indel; “.” = identity to reference sequence. Notes: The sequences in Figure S1 also can be found in the Supplementary Materials, file names: alignment of flanking sequence for Ptia5.fas, alignment of flanking sequence for Ptia2-intron.fas, and alignment for Ptia2-exon, which can be opened with software BioEdit version 7.0.2 [41].

## Abbreviations

The following abbreviations are used in this manuscript:

MCL	Maximum composite likelihood
STRs	Short tandem repeats
MYA	Million years ago
NGS	Next-generation sequencing
SNVs	Single-nucleotide variants
CSS	Cryptic simple sequences
SNPs	Single-nucleotide polymorphisms
ALT	*Panthera tigris altaica*
SCT	*Panthera tigris amoyensis*
