# Supplementary material for: Birth–Death Dynamics of Microsatellites: Mechanistic Insights from Orthologous Loci in Felidae
Source: Genes (Basel). 2025 Sep 19;16(9):1115. doi: 10.3390/genes16091115 (PMC12469459; doi:10.3390/genes16091115)
Supplement: Supplementary file 1 [file genes-16-01115-s001.zip › Figure S1.pdf]

```
                                            10        20
                                   ....|....|....|....|
Felis_catus                        AAGGAACTCCCTCAACAAAT
Felis_bieti                        ....................
Felis_silvestris                   ....................
Felis_nigripes                     ..........T.........
Felis_chaus                        ....................
Lynx_rufus                         ....................
Lynx_pardinus                      ....................
Lynx_canadensis                    ....................
Acinonyx_jubatus                   ....................
Neofelis_nebulosa                  ....................
Neofelis_diardi                    ....................
Prionailurus_bengalensis           ....................
Prionailurus_viverrinus            ....................
Prionailurus_iriomotensis          ....................
Panthera_leo                       ....................
Panthera_onca                      ....................
Panthera_pardus                    ..............G.....
Panthera_pardus_nimr               ..............G.....
Panthera_uncia                     ....................
Panthera_tigris_tigris             ....................
Panthera_tigris_jacksoni           ....................
Panthera_tigris_altaica            ....................
Panthera_tigris_amoyensis          ....................
Puma_concolor                      ....................
Puma_yagouaroundi                  ....................
Leopardus_geoffroyi                .G..................
Caracal_caracal                    ....................
Otocolobus_manul                   ....................
Catopuma_temmincki                 ....................
Hyaena_hyaena                      CCAA.G.CTG..TGTGTCT.
Crocuta_crocuta                    CCAA.G.CTG..TGTGTCT.
Proteles_cristata_cristata         CCAA.G.CTG..TGTGTCT.
Paguma_larvata                     CCAA.G..GG..TTTACCT.
Paradoxurus_hermaphroditus         CCAA.G..TG..TTTACCT.
Ailuropoda_melanoleuca             ..A.--..TG.CTTTGTCT.

                                            30        40
                                   ....|....|....|....|
Felis_catus                        ATTTATTAAGCATAATTTCT
Felis_bieti                        ....................
Felis_silvestris                   ....................
Felis_nigripes                     ....................
Felis_chaus                        ....................
Lynx_rufus                         ....................
```

```
Panthera_leo                    . . . . . . . . . . . . . . . . . . . .
Panthera_onca                   . . . . . . . . . . . . . . . . . . . .
Panthera_pardus                 . . . . . . . . . . . . . G . . . . . .
Panthera_pardus_nimr            . . . . . . . . . . . . . G . . . . . .
Panthera_uncia                  . . . . . . . . . . . . . . . . . . . .
Panthera_tigris_tigris          . . . . . . . . . . . . . . . . . . . .
Panthera_tigris_jacksoni        . . . . . . . . . . . . . . . . . . . .
Panthera_tigris_altaica         . . . . . . . . . . . . . . . . . . . .
Panthera_tigris_amoyensis       . . . . . . . . . . . . . . . . . . . .
Puma_concolor                   . . . . . . . . . . . . . . . . . . . .
Puma_yagouaroundi               . . . . . . . . . . . . . . . . . . . .
Leopardus_geoffroyi             . . . . . . . . . . . . . . . . . . . .
Caracal_caracal                 . . . . . . . . . . . . . . . . . . . .
Otocolobus_manul                . . . . . . . . . . . . . . . . . . . .
Catopuma_temmincki              . . . . . . . . . . . . . . . . . . . .
Hyaena_hyaena                   . . . . . . . . . G . . . . . . . A . .
Crocuta_crocuta                 . . . . . . . . . G . . . . . . . A . .
Proteles_cristata_cristata      . . . . . . . . . G . . . . . . . A . .
Paguma_larvata                  . . . . . . . . . G . . . . . . . A . .
Paradoxurus_hermaphroditus      . . . . . . . . . G . . . . . . . A . C
Ailuropoda_melanoleuca          . . T . . . . T . G . . T C A T . T C .

                                                  70                  80
                                . . . . | . . . . | . . . . | . . . . |
Felis_catus                     G - G T A T C C A A - G G A G A G A A A
Felis_bieti                     . - . . . . . . . . - . . . . . . . . .
Felis_silvestris                . - . . . . . . . . - . . . . . . . . .
Felis_nigripes                  . - . . . . . . . . - A . . A . . . . .
Felis_chaus                     . - . . . . . . . . - . . . . . . . . .
Lynx_rufus                      . - . . . . . . . . - . . . . C . . . .
Lynx_pardinus                   . - . . . . . . . . - . . . . C . . . .
Lynx_canadensis                 . - . . . . . . . . - . . . . C . . . .
Acinonyx_jubatus                . - . . . . . . . . - . . . . C . . . .
Neofelis_nebulosa               . - . . . . . . . . - C . . . C . . . .
Neofelis_diardi                 . - . . . . . . . . - C . . . C . . . .
Prionailurus_bengalensis        . - . . . . . . . . - . . . . . . . . .
Prionailurus_viverrinus         . - . . . . . . . . - . . . . . . . . .
Prionailurus_iriomotensis       . - . . . . . . . . - . . . . . . . . .
Panthera_leo                    . - . . . . . . . . - . . . . C . . . .
Panthera_onca                   . - . . . . . . . . - . . . . C . . . .
Panthera_pardus                 . - . . . . . . . . - . . . . C . . . .
Panthera_pardus_nimr            . - . . . . . . . . - . . . . C . . . .
Panthera_uncia                  . - . . . . . . . . - C . . . C . . . .
Panthera_tigris_tigris          . - . . . . . . . . - C . . . C . . . .
Panthera_tigris_jacksoni        . - . . . . . . . . - C . . . C . . . .
Panthera_tigris_altaica         . - . . . . . . . . - C . . . C . . . .
```

```
Panthera_tigris_amoyensis        . - . . . . . . . . - C . . . C . . . .
Puma_concolor                    . - . . . . . . . . - . . . . C . . . .
Puma_yagouaroundi                . - . . . . . . . . - . . . . C . . . .
Leopardus_geoffroyi              . - . . . . . . . . - . . . . C . . . .
Caracal_caracal                  . - . . . . . . . . - . . . . C . . . .
Otocolobus_manul                 . - . . . . . . . . - . . . . T . . . .
Catopuma_temmincki               . - . . . . . . . . - . . . . T . . . .
Hyaena_hyaena                    . - . . . . . T . . - . A . . T . . . .
Crocuta_crocuta                  . - . . . . . T . . - . A . . C . . . .
Proteles_cristata_cristata       . - A . . . . T . . - . A . . C A . . .
Paguma_larvata                   . - . . . . . T . . - . A . . C A . . G
Paradoxurus_hermaphroditus       . - . . . . . T . . - . A C . C A . . G
Ailuropoda_melanoleuca           A A . . . . . T . A . A . . C T . . . .

                                                   90                  100
                                 . . . . | . . . . | . . . . | . . . . |
Felis_catus                      T G G A A T A T T C A A A G G A A C A G
Felis_bieti                      . . . . . . . . . . . . . . A . . . . .
Felis_silvestris                 . . . . . . . . . . . . . . . . . . . .
Felis_nigripes                   . . . . . . . . . . . . . . . . . . . .
Felis_chaus                      . . . . . . . . . . . . . . . . . . . .
Lynx_rufus                       . . . . . . . . . . . . . . . . . . . .
Lynx_pardinus                    . . . . . . . . . . . . . . . . . G . .
Lynx_canadensis                  . . . . . . . . . . . . . . . . . G . .
Acinonyx_jubatus                 . . . . . . . . . . . . . . . . . . . .
Neofelis_nebulosa                . . . . . . . . . . . . . . . . . . . .
Neofelis_diardi                  . . . . . . . . . . . . . . . . . . . .
Prionailurus_bengalensis         . . . . . . . . . . . . . . . . . . . .
Prionailurus_viverrinus          . . . . . . . . . . . . . . . . . . . .
Prionailurus_iriomotensis        . . . . . . . . . . . . . . . . . . . .
Panthera_leo                     . . . . . . . . . . . . . . . . . . . .
Panthera_onca                    . . . . . . . . . . . . . . . . . . . .
Panthera_pardus                  . . . . . . . . . . . . . . . . . . . .
Panthera_pardus_nimr             . . . . . . . . . . . . . . . . . . . .
Panthera_uncia                   . . . . . . . . . . . . . . . . . . . .
Panthera_tigris_tigris           . . . . . . . . . . . . . . . . . . . .
Panthera_tigris_jacksoni         . . . . . . . . . . . . . . . . . . . .
Panthera_tigris_altaica          . . . . . . . . . . . . . . . . . . . .
Panthera_tigris_amoyensis        . . . . . . . . . . . . . . . . . . . .
Puma_concolor                    . . . . . . . . . . . . . . . . . . . .
Puma_yagouaroundi                . . . . . . . . . . . . . . . . . . . .
Leopardus_geoffroyi              . . . . . . . . . . G . . . . . . . . .
Caracal_caracal                  . . . . . . . . . . . . . . . . . . . .
Otocolobus_manul                 . . . . . . . . . . . . . . . . . . . .
Catopuma_temmincki               . . . . . . . . . . . . . . . . . . . .
Hyaena_hyaena                    . . . . T . . . . . . . . . . . . . . .
```

```
Crocuta_crocuta                . . . . T . . . . . . . . . . . . . . .
Proteles_cristata_cristata     . A . . T . . . . . . . . . . . . . . .
Paguma_larvata                 . . . . T . . . . . . . . . . . . . . .
Paradoxurus_hermaphroditus     . . . . T . . . . . . . G . . . . . . .
Ailuropoda_melanoleuca         . A . . T . . . . T . . G . . . . . . .

                                                 110                 120
                               . . . . | . . . . | . . . . | . . . . |
Felis_catus                    A G C T C C T T G - G G G G A G C A G G
Felis_bieti                    . . . . . . . . . - . . . . . . . . . .
Felis_silvestris               . . . . . . . . . - . . . . . . . . . .
Felis_nigripes                 . . . . . . . . . - . . . . . . . . . .
Felis_chaus                    . . . . . . . . . - . . . T . . . . . .
Lynx_rufus                     . . . . . . . . . - . . . T . . . . . .
Lynx_pardinus                  . . . . . . . . . - . . . T . . . . . .
Lynx_canadensis                . . . . . . . . . - . . . T . . . . . .
Acinonyx_jubatus               . . . . . . . . . - . . . T . . - . . .
Neofelis_nebulosa              . . . . . . . . . - . . . T . . . . . .
Neofelis_diardi                . . . . . . . . . - . . . T . . . . . .
Prionailurus_bengalensis       . . . . . . . . . - . . . T . . . . . .
Prionailurus_viverrinus        . . . . . . . . . - . . . T . . . . . .
Prionailurus_iriomotensis      . . . . . . . . . - . . . T . . . . . .
Panthera_leo                   . . . . . . . . . - . . . T . . . . . .
Panthera_onca                  . . . . . . . . . - . - - - - - . . . .
Panthera_pardus                . . . . . . . . . - . . . T . . . . . .
Panthera_pardus_nimr           . . . . . . . . . - . . . T . . . . . .
Panthera_uncia                 . . . . . . . . . - . . . T . . . . . .
Panthera_tigris_tigris         . . . . . . . . . - . . . T . . . . . .
Panthera_tigris_jacksoni       . . . . . . . . . - . . . T . . . . . .
Panthera_tigris_altaica        . . . . . . . . . - . . . T . . . . . .
Panthera_tigris_amoyensis      . . . . . . . . . - . . . T . . . . . .
Puma_concolor                  . . . . . . . . . - . . . T . . - . . .
Puma_yagouaroundi              . . . . . . . . . - . . . T . . - . . .
Leopardus_geoffroyi            . . . . . . . . . - . . . T . . . . . .
Caracal_caracal                . . . . . . . . . - . . . T . . . . . .
Otocolobus_manul               . . . . . . . . . - . . . T . . . . . .
Catopuma_temmincki             . . . . . . . . . - . . . T . . . . . .
Hyaena_hyaena                  . . . . T . . . . - . . T T . . . . . .
Crocuta_crocuta                . . . . T . . . . - . . T T . . . . . .
Proteles_cristata_cristata     . . . . T . . . . - . . T T . . . . . .
Paguma_larvata                 T . . . . . C . C C A . T T . . T . . .
Paradoxurus_hermaphroditus     T . . . . . . . C C A . T T . . T . . .
Ailuropoda_melanoleuca         . - . C . . . . . - - T T T . . T . . .

                                                 130                 140
                               . . . . | . . . . | . . . . | . . . . |
```

```
Felis_catus                      G T T C T T T A C T C C C C T G G C T G
Felis_bieti                      . . . . . . . . . . . . . . . . . . . .
Felis_silvestris                 . . . . . . . . . . . . . . . . . . . .
Felis_nigripes                   . . . . . . . . . . . . . T . . . . . .
Felis_chaus                      . . . . . . . . . . . . . . . . . . . .
Lynx_rufus                       . . . . . . . . . . . . . . . . . . . .
Lynx_pardinus                    . . . . . . . . . . . . . . . . . . . .
Lynx_canadensis                  . . . . . . . . . . . . . . . . . . . .
Acinonyx_jubatus                 . . . . . . . . . . . . . . . . . . . .
Neofelis_nebulosa                . . . . . . . . . . . . . . . . . . . .
Neofelis_diardi                  . . . . . . . . . . . . . . . . . . . .
Prionailurus_bengalensis         . . . . . . . . G . . . . . . . . . . .
Prionailurus_viverrinus          . . . . . . . . G . . . . . . . . . . .
Prionailurus_iriomotensis        . . . . . . . . G . . . . . . . . . . .
Panthera_leo                     . . . . . . . . . . . . . . . . . . . .
Panthera_onca                    . . . . . . . . . . . . . . . . . . . .
Panthera_pardus                  . . . . . . . . . . . . . . . . . . . .
Panthera_pardus_nimr             . . . . . . . . . . . . . . . . . . . .
Panthera_uncia                   . . . . . . . C T . . . . . . . . . . .
Panthera_tigris_tigris           . . . . . . . C . . . . . . . . . . . .
Panthera_tigris_jacksoni         . . . . . . . C . . . . . . . . . . . .
Panthera_tigris_altaica          . . . . . . . C . . . . . . . . . . . .
Panthera_tigris_amoyensis        . . . . . . . C . . . . . . . . . . . .
Puma_concolor                    . . . . . . . . . . . . . . . . . . . .
Puma_yagouaroundi                . . . . . . . . . . . . . . . . . . . .
Leopardus_geoffroyi              . . . . . . . . . . . . . . . . . . . .
Caracal_caracal                  . K . . . . . . . . . . . . C . . . . .
Otocolobus_manul                 . . . . . . . . . . . . . . . . . . . .
Catopuma_temmincki               . . . . . . . . . . . . . . . . . . . .
Hyaena_hyaena                    . . . . . . . G . . . . . . . . A . . .
Crocuta_crocuta                  . . . . . . . G . . . . . . . . A . . .
Proteles_cristata_cristata       . . . . . . . . . . . . . . . . A . . .
Paguma_larvata                   T . . . . . . . . . . . . . . . A . . .
Paradoxurus_hermaphroditus       T . . . . . . . . . . . . . . . A . . .
Ailuropoda_melanoleuca           T . . . . . . . . . . . . . C A . . . .

                                                   150                 160
                                 . . . . | . . . . | . . . . | . . . . |
Felis_catus                      A T A A C A G G T A G A G G T G A C C A
Felis_bieti                      . . . . . . . . . . . . . . . . . . . .
Felis_silvestris                 . . . . . . . . . . . . . . . . . . . .
Felis_nigripes                   . . . . . . . . . . . . . . . . . . . .
Felis_chaus                      . . . . . . . . . . . . . . . . . . . .
Lynx_rufus                       . . . . . . . A . . . . . . . . . . . .
Lynx_pardinus                    . . . . . . . A . . . . . . . . . . . .
Lynx_canadensis                  . . . . . . . A . . . . . . . . . . . .
```

```
Acinonyx_jubatus               . . . . . . . . . . . . . . . . . . . .
Neofelis_nebulosa              . . . . . . . . . . . . . . . . . . . .
Neofelis_diardi                . . . . . . . . . . . . . . . . . . . .
Prionailurus_bengalensis       . . . . . . . . . . . . . . . . . . . .
Prionailurus_viverrinus        . . . . . . . . . . . . . . . . . . . .
Prionailurus_iriomotensis      . . . . . . . . . . . . . . . . . . . .
Panthera_leo                   . . . . . . . . . . . . . . . . . . . .
Panthera_onca                  . . . . . . . . . . . . . . . . . . . .
Panthera_pardus                . . . . . . . . . . . . . . . . . . . .
Panthera_pardus_nimr           . . . . . . . . . . . . . . . . . . . .
Panthera_uncia                 . . . . . . . . . . . . . . . . . . . .
Panthera_tigris_tigris         . . . . . . . . . . . . . . . . . . . .
Panthera_tigris_jacksoni       . . . . . . . . . . . . . . . . . . . .
Panthera_tigris_altaica        . . . . . . . . . . . . . . . . . . . .
Panthera_tigris_amoyensis      . . . . . . . . . . . . . . . . . . . .
Puma_concolor                  . . . . . . . . . . . . . . . . . . . .
Puma_yagouaroundi              . . . . . . . . . . . . . . . . . . . .
Leopardus_geoffroyi            . . . . . . . . . . . . . . . . . . . .
Caracal_caracal                . . . . . . . . . . . . . . . . . . . .
Otocolobus_manul               . . . . . . . . . . . . . . . . . . . .
Catopuma_temmincki             . . . . . . . . . . . . . . . . . . . .
Hyaena_hyaena                  . A . . . . . . . . . . . . . . . . . .
Crocuta_crocuta                . A . . . . . . . . . . . . . . . . . G
Proteles_cristata_cristata     . A . . . . . . . . . . . . . . . . . .
Paguma_larvata                 . . . . . . . . . . . . . . . . . . . .
Paradoxurus_hermaphroditus     . . . . . . . . . . . . . . . . . . . .
Ailuropoda_melanoleuca         . C . C . . . . . . . . . . . . . . . .

                                                 170                 180
                               . . . . | . . . . | . . . . | . . . . |
Felis_catus                    T C A C C T T T A G G G A C A G A A G G
Felis_bieti                    . . . . . . . . C . . . . . . . . . . .
Felis_silvestris               . . . . . . . . . . . . . . . . . . . .
Felis_nigripes                 . . . . . . . . . . . . . . . . . . . .
Felis_chaus                    . . . . . . . . . . . . . . . . . . . .
Lynx_rufus                     . . . . . . . . . . . . G A . . . . . .
Lynx_pardinus                  . . . . . . . . . . . . G . . . . . . .
Lynx_canadensis                . . . . . . . . . . . . G . . . . . . .
Acinonyx_jubatus               . . . . . . . . . . . . . . . . . . . .
Neofelis_nebulosa              . . . . . . . . . . . . . . . . . . . .
Neofelis_diardi                . . . . . . . . . . . . . . . . . . . .
Prionailurus_bengalensis       . . . . . . . . . . . . . . . . . . . .
Prionailurus_viverrinus        . . . . . . . . . . . . . . . . . . . .
Prionailurus_iriomotensis      . . . . . . . . . . . . . . . . . . . .
Panthera_leo                   . . . . . . . . . . . . . . . . . . . .
Panthera_onca                  . . . . . . . . . . . . . . . . . . . .
```

```
Panthera_pardus                     . . . . . . . . . . . . . . . . . C . .
Panthera_pardus_nimr                . . . . . . . . . . . . . . . . . C . .
Panthera_uncia                      . . . . . . . . . . . . . . . . . . . .
Panthera_tigris_tigris              . . . . . . . . . . . . . . . . . . . .
Panthera_tigris_jacksoni            . . . . . . . . . . . . . . . . . . . .
Panthera_tigris_altaica             . . . . . . . . . . . . . . . . . . . .
Panthera_tigris_amoyensis           . . . . . . . . . . . . . . . . . . . .
Puma_concolor                       . . . . . . . . . . . . . . . . . . . .
Puma_yagouaroundi                   . . . . . . . . . . . . . . . . . . . .
Leopardus_geoffroyi                 . . . . . . . . . . . . G . . . . . . .
Caracal_caracal                     . . . . . . . . . . . . G . . . . . . .
Otocolobus_manul                    . . . . . . . . . . . . . . . . . . . .
Catopuma_temmincki                  . . G . T . . . - - - - - - - - - - - -
Hyaena_hyaena                       . . . A . . . . . A . . . . . . . . . .
Crocuta_crocuta                     . . . A . . . . . A . . . . . . . . . .
Proteles_cristata_cristata          . . . A . . . . . A . . . . . . . . . .
Paguma_larvata                      . . . . . . . . . . . . . . . . . . . .
Paradoxurus_hermaphroditus          . . . . . . . . . . . . . . . . . . . .
Ailuropoda_melanoleuca              . T . . . . . . . . T . . . . . . . A A

                                                      190                 200
                                    . . . . | . . . . | . . . . | . . . . |
Felis_catus                         A C A G C T A T G C A G A G C T C A T T
Felis_bieti                         . . . . . . . . . . . . . . . . . . . .
Felis_silvestris                    . . . . . . . . . . . . . . . . . . . .
Felis_nigripes                      . . . . . . . . . . . . . . . . . . . .
Felis_chaus                         . . . . . . . . . . . . . . . . . . . .
Lynx_rufus                          . . . . . . . . . . . . . . . . . G . .
Lynx_pardinus                       . . . . . . . . . . . . . . . . . T . .
Lynx_canadensis                     . . . . . . . . . . . . . . . . . G . .
Acinonyx_jubatus                    . . . . . . . . . . . . . . . . . G . .
Neofelis_nebulosa                   . . . . . . . . . . . . . . . . . G . .
Neofelis_diardi                     . . . . . . . . . . . . . . . . . G . .
Prionailurus_bengalensis            . . . . . . . . . . . . . . . . . G . .
Prionailurus_viverrinus             . . . . . . . . . . . . . . . . . G . .
Prionailurus_iriomotensis           . . . . . . . . . . . . . . . . . G . .
Panthera_leo                        . . . . . . . . . . . . . . . . . G . .
Panthera_onca                       . . . . . . . . . . . . . . . . . G . .
Panthera_pardus                     . . . . . . . . . . . . . . . . . G . .
Panthera_pardus_nimr                . . . . . . . . . . . . . . . . . G . .
Panthera_uncia                      . . . . . . . . . . . . . . . . . G . .
Panthera_tigris_tigris              . . . . . . . . . . . . . . . . . G . .
Panthera_tigris_jacksoni            . . . . . . . . . . . . . . . . . G . .
Panthera_tigris_altaica             . . . . . . . . . . . . . . . . . G . .
Panthera_tigris_amoyensis           . . . . . . . . . . . . . . . . . G . .
Puma_concolor                       . . . . . . . . . . . . . . . . . G . .
```

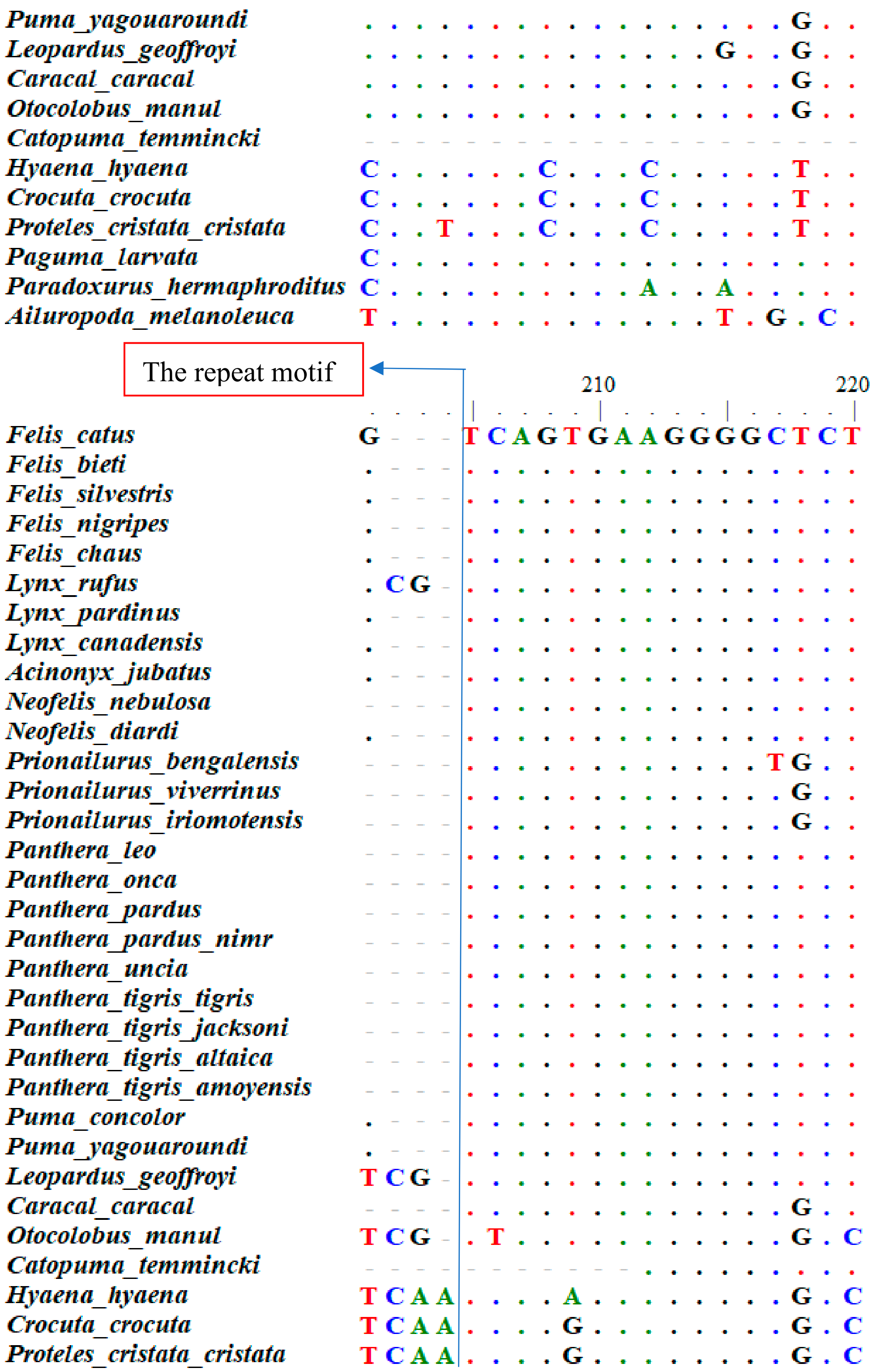
Puma_yagouaroundi
Leopardus_geoffroyi
Caracal_caracal
Otocolobus_manul
Catopuma_temmincki
Hyaena_hyaena
Crocuta_crocuta
Proteles_cristata_cristata
Paguma_larvata
Paradoxurus_hermaphroditus
Ailuropoda_melanoleuca
The repeat motif
210
220
Felis_catus
G - - - T C A G T G A A G G G G C T C T
Felis_bieti
Felis_silvestris
Felis_nigripes
Felis_chaus
Lynx_rufus
Lynx_pardinus
Lynx_canadensis
Acinonyx_jubatus
Neofelis_nebulosa
Neofelis_diardi
Prionailurus_bengalensis
Prionailurus_viverrinus
Prionailurus_iriomotensis
Panthera_leo
Panthera_onca
Panthera_pardus
Panthera_pardus_nimr
Panthera_uncia
Panthera_tigris_tigris
Panthera_tigris_jacksoni
Panthera_tigris_altaica
Panthera_tigris_amoyensis
Puma_concolor
Puma_yagouaroundi
Leopardus_geoffroyi
Caracal_caracal
Otocolobus_manul
Catopuma_temmincki
Hyaena_hyaena
Crocuta_crocuta
Proteles_cristata_cristata

```
Paguma_larvata                TCAT|....G..G...A.G.C
Paradoxurus_hermaphroditus    TCAT|..G.G.CG...A.G.C
Ailuropoda_melanoleuca        TCA-|-TGAAATGTCTA..T.
```

```
                                        230
                              ....|....|....
Felis_catus                   TGGGTGGCTCAGTC
Felis_bieti                   ..............
Felis_silvestris              ..............
Felis_nigripes                ..............
Felis_chaus                   ..............
Lynx_rufus                    ..............
Lynx_pardinus                 ..............
Lynx_canadensis               ..............
Acinonyx_jubatus              ..............
Neofelis_nebulosa             ..............
Neofelis_diardi               ..............
Prionailurus_bengalensis      ..............
Prionailurus_viverrinus       ..............
Prionailurus_iriomotensis     ..............
Panthera_leo                  ..............
Panthera_onca                 ..............
Panthera_pardus               ..............
Panthera_pardus_nimr          ..............
Panthera_uncia                ..............
Panthera_tigris_tigris        ..............
Panthera_tigris_jacksoni      ..............
Panthera_tigris_altaica       ..............
Panthera_tigris_amoyensis     ..............
Puma_concolor                 ..............
Puma_yagouaroundi             ..............
Leopardus_geoffroyi           ..............
Caracal_caracal               ..............
Otocolobus_manul              ..............
Catopuma_temmincki            ..............
Hyaena_hyaena                 ......TT.....G
Crocuta_crocuta               ......TT.....G
Proteles_cristata_cristata    ......TT...A.G
Paguma_larvata                ....G........T
Paradoxurus_hermaphroditus    ....G........T
Ailuropoda_melanoleuca        .CACGAAG...A.A
```

a

	10 20
	....|....|....|....|
Panthera_tigris_tigris	AGTTGGGAATG--CTGCTCT
Felis_catus	...........--.......
Felis_chaus	...........--.......
Felis_nigripes	...........--.......
Felis_silvestris	...........--.......
Felis_bieti	...........--.......
Otocolobus_manul	...........--.......
Prionailurus_bengalensis	...........--.......
Prionailurus_bengalensis_eu	...........--.......
Prionailurus_iriomotensis	...........--.......
Prionailurus_viverrinus	.A.........--.......
Acinonyx_jubatus	...........--.......
Puma_concolor	...........--.......
Puma_yagouaroundi	...........--...T...
Lynx_canadensis	...........--.......
Lynx_pardinus	...........--.......
Lynx_rufus	...........--.......
Leopardus_geoffroyi	...........--.......
Caracal_caracal	...........--.......
Catopuma_temminckii	...........--.......
Neofelis_nebulosa	...........--.......
Neofelis_diardi	...........--.......
Neovison_vison	.......G...--.......
Panthera_tigris_jacksoni	...........--.......
Panthera_tigris_altaica	...........--.......
Panthera_leo	...........--.......
Panthera_onca	...........--.......
Panthera_pardus	...........--.......
Panthera_pardus_nimr	...........--.......
Panthera_uncia	...........--.......
Panthera_tigris_amoyensis	...........--.......
Crocuta_crocuta	G....A.....TG.......
Hyaena_hyaena	G....A.....TG.......
Proteles_cristata_cristata	G....A.....TG.......
Paradoxurus_hermaphroditus	..........ATG.C.....
Potos_flavus	.......G..A--.....G.
Paguma_larvata	..........ATG.C.....
Ailuropoda_melanoleuca	.......G...--.......

	30 40
	....|....|....|....|
Panthera_tigris_tigris	GTGAGACAATTAGCAGGATG
Felis_catus	....
Felis_chaus	....

```
Felis_nigripes                       ....................
Felis_silvestris                     ....................
Felis_bieti                          ....................
Otocolobus_manul                     ....................
Prionailurus_bengalensis             ....................
Prionailurus_bengalensis_eu          ....................
Prionailurus_iriomotensis            ....................
Prionailurus_viverrinus              ....................
Acinonyx_jubatus                     ....................
Puma_concolor                        ....................
Puma_yagouaroundi                    ....................
Lynx_canadensis                      ....................
Lynx_pardinus                        ....................
Lynx_rufus                           ....................
Leopardus_geoffroyi                  ....................
Caracal_caracal                      ....................
Catopuma_temminckii                  ....................
Neofelis_nebulosa                    ....................
Neofelis_diardi                      ....................
Neovison_vison                       C......G..A.A..A....
Panthera_tigris_jacksoni             ....................
Panthera_tigris_altaica              ....................
Panthera_leo                         ....................
Panthera_onca                        ....................
Panthera_pardus                      ....................
Panthera_pardus_nimr                 ....................
Panthera_uncia                       ....................
Panthera_tigris_amoyensis            ....................
Crocuta_crocuta                      C.........A....A....
Hyaena_hyaena                        C.........A....A....
Proteles_cristata_cristata           C.........A....A....
Paradoxurus_hermaphroditus           C........GA....A....
Potos_flavus                         C.........----.A....
Paguma_larvata                       C........GA....A....
Ailuropoda_melanoleuca               C.........A....A....

                                              50        60
                                     ....|....|....|....|
Panthera_tigris_tigris               GGTAAAAACCCCAAT-----
Felis_catus                          ...............-----
Felis_chaus                          ...............-----
Felis_nigripes                       ...............-----
Felis_silvestris                     ...............-----
Felis_bieti                          .......-.......-----
Otocolobus_manul                     ...............-----
Prionailurus_bengalensis             ...............-----
```

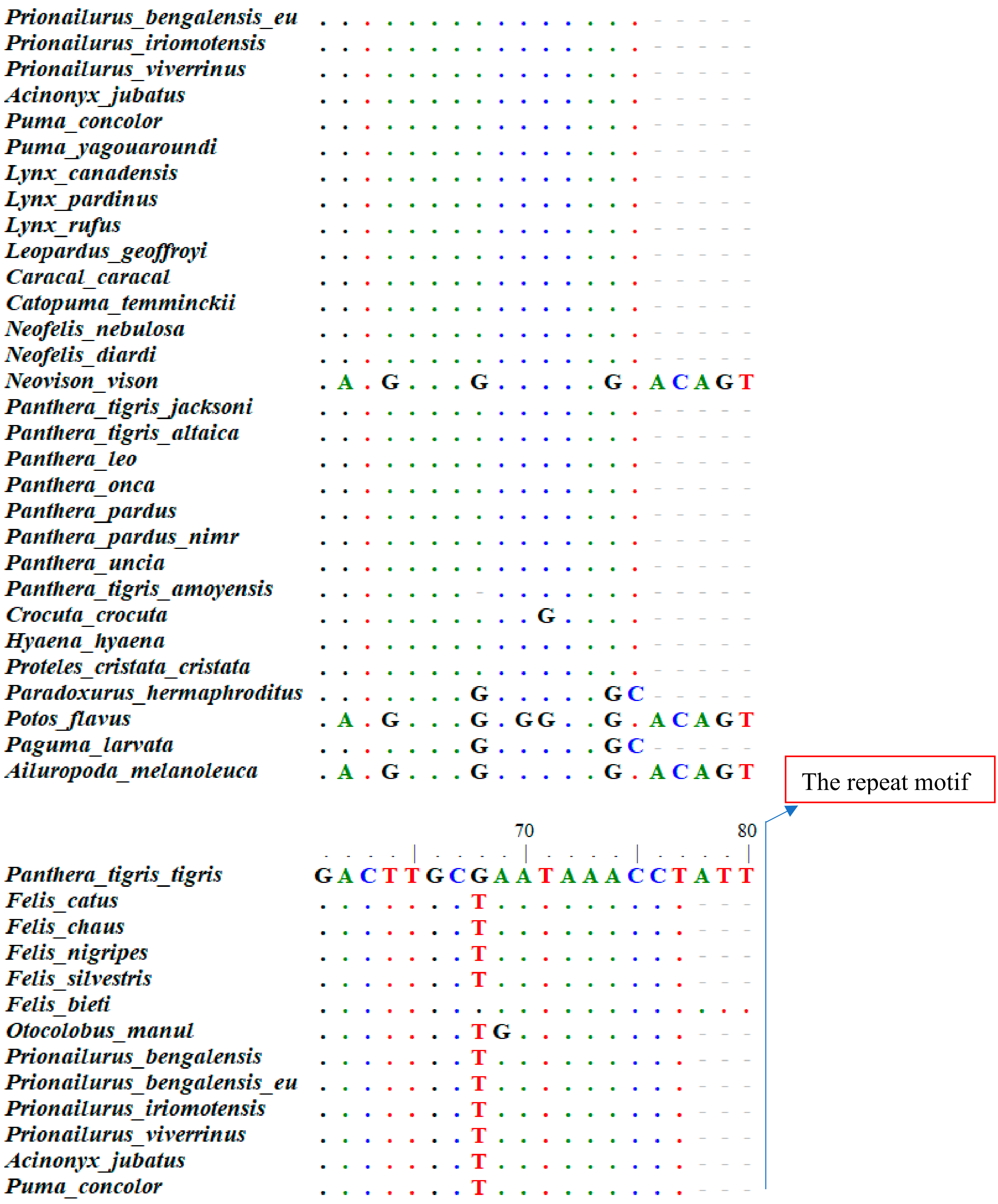
Prionailurus_bengalensis_eu
Prionailurus_iriomotensis
Prionailurus_viverrinus
Acinonyx_jubatus
Puma_concolor
Puma_yagouaroundi
Lynx_canadensis
Lynx_pardinus
Lynx_rufus
Leopardus_geoffroyi
Caracal_caracal
Catopuma_temminckii
Neofelis_nebulosa
Neofelis_diardi
Neovison_vison
Panthera_tigris_jacksoni
Panthera_tigris_altaica
Panthera_leo
Panthera_onca
Panthera_pardus
Panthera_pardus_nimr
Panthera_uncia
Panthera_tigris_amoyensis
Crocuta_crocuta
Hyaena_hyaena
Proteles_cristata_cristata
Paradoxurus_hermaphroditus
Potos_flavus
Paguma_larvata
Ailuropoda_melanoleuca
The repeat motif
70
80
Panthera_tigris_tigris
GACTTGCGAATAAACCTATT
Felis_catus
Felis_chaus
Felis_nigripes
Felis_silvestris
Felis_bieti
Otocolobus_manul
Prionailurus_bengalensis
Prionailurus_bengalensis_eu
Prionailurus_iriomotensis
Prionailurus_viverrinus
Acinonyx_jubatus
Puma_concolor

```
Puma_yagouaroundi             . . . . . . . T . . . . . . . . . - - -
Lynx_canadensis               . . . . . . . T . . . . . . . . . - - -
Lynx_pardinus                 . . . . . . . T . . . . . . . . . - - -
Lynx_rufus                    . . . . . . . T . . . . . . . . . - - -
Leopardus_geoffroyi           . . . . . . . T . . . . . . . . . - - -
Caracal_caracal               . . . . . . . T . . . . . . . . . - - -
Catopuma_temminckii           . . . . . . . T . . . . . . . . . - - -
Neofelis_nebulosa             . . . . . . . T . . . . . . . . . - - -
Neofelis_diardi               . . . . . . . T . . . . . . . . . - - -
Neovison_vison                . . . . . . . T . . . . C . . A . T A C
Panthera_tigris_jacksoni      . . . . . . . . . . . . . . . . . . . .
Panthera_tigris_altaica       . . . . . . . . . . . . . . . . . . . .
Panthera_leo                  . . . . . . . . . . . . . . . . . . . .
Panthera_onca                 . . . . . . . . . . . . . . . . . . . .
Panthera_pardus               . . . . . . . . . . . . . . . . . . . .
Panthera_pardus_nimr          . . . . . . . . . . . . . . . . . . . .
Panthera_uncia                . . . . . . . . . . . . . . . . . . . .
Panthera_tigris_amoyensis     . . . . . . . . . . . . . . . . . . . .
Crocuta_crocuta               . . . . . A . T . . . . . . . . . - - -
Hyaena_hyaena                 . . . G . A . T . . . . . . . . . - - -
Proteles_cristata_cristata    . . . . . A . T . . . . . . . . . G A .
Paradoxurus_hermaphroditus    . . . . . . . T . . . . . . . . . G C A
Potos_flavus                  . . . . . . . T . G . . . . . A . - - -
Paguma_larvata                . . . . . . . T . . . . . . . . . G C A
Ailuropoda_melanoleuca        . . . . . . . T . G . . . . T G . - - -

                                                90                 100
                              . . . . | . . . . | . . . . | . . . . |
Panthera_tigris_tigris        - - - - G C G T A T T G T T A G A G G G
Felis_catus                   - - - - . . . . . . . . . . . . . . . .
Felis_chaus                   - - - - . . . . . . . . . . . . . . . .
Felis_nigripes                - - - - . . . . . . . . . . . . . . . .
Felis_silvestris              - - - - . . . . . . . . . . . . . . . .
Felis_bieti                   - - - - . . . . . . . . . . . . . . . .
Otocolobus_manul              - - - - . T . . . . . . . . . . . . . .
Prionailurus_bengalensis      - - - - . T . . . . . . . . . . . . . .
Prionailurus_bengalensis_eu   - - - - . T . . . . . . . . . . . . . .
Prionailurus_iriomotensis     - - - - . T . . . . . . . . . . . . . .
Prionailurus_viverrinus       - - - - . T . . . . . . . . . . . . . .
Acinonyx_jubatus              - - - - . T . . . . . . . . . . . . . .
Puma_concolor                 - - - - . T . . . . . . . . . . . . . .
Puma_yagouaroundi             - - - - . T . . . . . . . . . . . . . .
Lynx_canadensis               - - - - . T . . . . . . . . . . . . . .
Lynx_pardinus                 - - - - . T . . . . . . . . . . . . . .
Lynx_rufus                    - - - - . T . . . . . . . . . . . . . .
Leopardus_geoffroyi           - - - - . . . . . . . . . . . . . . . .
```

```
Caracal_caracal                 - - - - . . . . . . . . . . . . . . . .
Catopuma_temminckii             - - - - . . . . . . . . . . . . . . . .
Neofelis_nebulosa               - - - - . T . . . . . . . . . . . . . .
Neofelis_diardi                 - - - - . T . . . . . . . . . . . . . .
Neovison_vison                  A T G C T G T C . . . . . . . . . . . .
Panthera_tigris_jacksoni        - - - - . . . . . . . . . . . . . . . .
Panthera_tigris_altaica         - - - - . . . . . . . . . . . . . . . .
Panthera_leo                    - - - - . . . . . . . . . . . . . . . .
Panthera_onca                   - - - - . . . . . . . . . . . . . . . .
Panthera_pardus                 - - - - . . A . . . . . . . . . . . . .
Panthera_pardus_nimr            - - - - . . . . . . . . . . . . . . . .
Panthera_uncia                  - - - - . . . . . . . . . . . . . . . .
Panthera_tigris_amoyensis       - - - - . . . . . . . . . . . . . . . .
Crocuta_crocuta                 - - - - A . A . . . . . . . . . . . . .
Hyaena_hyaena                   - - - - A . A . . . . . . . . . . . . .
Proteles_cristata_cristata      - - - - A . A . . . . . . . . . . . . .
Paradoxurus_hermaphroditus      - - - - . . . . . . C . . . . . C . . .
Potos_flavus                    - - - - - G T . . . . . . . . . . . . .
Paguma_larvata                  - - - - . . . . . . . . . . . . C . . .
Ailuropoda_melanoleuca          - - - - - G T . . C . . . . . . . . . .

                                                  110                 120
                                . . . . | . . . . | . . . . | . . . . |
Panthera_tigris_tigris          A A C T C A A C A G G A C A G A C T T C
Felis_catus                     . . . . . . . G . . . . . . . . . . . .
Felis_chaus                     . . . . . . . G . . . . . . . . . . . .
Felis_nigripes                  . . . . . . . G . . . . . . . . . . . .
Felis_silvestris                . . . . . . . . . . . . . . . . . . . .
Felis_bieti                     . . . . . . . . . . . . . . . . . . . .
Otocolobus_manul                . . . . . . . G . . . . . . . . . . . .
Prionailurus_bengalensis        . . . . . . . G . . . . . . . . . . . T
Prionailurus_bengalensis_eu     . . . . . . . G . . . . . . . . . . . T
Prionailurus_iriomotensis       . . . . . . . G . . . . . . . . . . . T
Prionailurus_viverrinus         . . . . . . . G . . . . . . . . . . . .
Acinonyx_jubatus                . . . . . . . G . . . . . . . . . . . .
Puma_concolor                   . . . . . . . G . . . . . . . . . . . .
Puma_yagouaroundi               . . . . . . . G . . . . . . . . . . . .
Lynx_canadensis                 . . . . . . . G . . . . . . . . . . . .
Lynx_pardinus                   . . . . . . . G . . . . . . . . . . . .
Lynx_rufus                      . . . . . . . G . . . . . . . . . . . .
Leopardus_geoffroyi             . . . . . . . G . . . . . . . . . . . .
Caracal_caracal                 . . . . . . . G . . . . . . . . . . . T
Catopuma_temminckii             . . . . . . . . . . . . . . . . . . . .
Neofelis_nebulosa               . . . . . . . . . . . . . . . . . . . .
Neofelis_diardi                 . . . . . . . . . . . . . . . . . . . .
Neovison_vison                  . . . . . . . G . . . . . . . G T . . .
```

Panthera_tigris_jacksoni	....
Panthera_tigris_altaica	....
Panthera_leo	....
Panthera_onca	....
Panthera_pardus	....
Panthera_pardus_nimr	....
Panthera_uncia	....
Panthera_tigris_amoyensis	....
Crocuta_crocuta	.......G........T...
Hyaena_hyaena	.......G........T...
Proteles_cristata_cristata	.......G........T...
Paradoxurus_hermaphroditus	.....--T........T...
Potos_flavus	.......G........T...
Paguma_larvata	.....--G........T...
Ailuropoda_melanoleuca	.......G..G.....T...

	....
Panthera_tigris_tigris	-GGA
Felis_catus	-...
Felis_chaus	-...
Felis_nigripes	-...
Felis_silvestris	-...
Felis_bieti	-...
Otocolobus_manul	-...
Prionailurus_bengalensis	-...
Prionailurus_bengalensis_eu	-...
Prionailurus_iriomotensis	-...
Prionailurus_viverrinus	-...
Acinonyx_jubatus	-...
Puma_concolor	-...
Puma_yagouaroundi	-...
Lynx_canadensis	-...
Lynx_pardinus	-...
Lynx_rufus	-...
Leopardus_geoffroyi	-...
Caracal_caracal	-...
Catopuma_temminckii	-...
Neofelis_nebulosa	-...
Neofelis_diardi	-...
Neovison_vison	T...
Panthera_tigris_jacksoni	-...
Panthera_tigris_altaica	-...
Panthera_leo	-...
Panthera_onca	-...
Panthera_pardus	-...

Panthera_pardus_nimr	-	A	.	.
Panthera_uncia	-	.	.	.
Panthera_tigris_amoyensis	-	.	.	.
Crocuta_crocuta	A	.	.	.
Hyaena_hyaena	A	.	.	.
Proteles_cristata_cristata	A	.	.	.
Paradoxurus_hermaphroditus	T	.	A	.
Potos_flavus	T	.	.	.
Paguma_larvata	T	.	A	.
Ailuropoda_melanoleuca	T	.	.	.

b

```
                                            10                  20
                                  . . . . | . . . . | . . . . | . . . . |
Felis_catus                       C T A G T A A A T G T T C C G G A G G A
Felis_chaus                       . . . . . . . . . . . . . . . . . . . .
Felis_nigripes                    . . . . . . . . . . . . . . . . . . . .
Otocolobus_manul                  . . . . . . . . . . . . . . . . . . . .
Prionailurus_bengalensis          . . . . . . . . . . . . . . . . . . . .
Prionailurus_bengalensis_eu       . . . . . . . . . . . . . . . . . . . .
Prionailurus_iriomotensis         . . . . . . . . . . . . . . . . . . . .
Prionailurus_viverrinus           . . . . . . . . . . . . . . . . . . . .
Acinonyx_jubatus                  . . . . . . . . . . . . . . . . . . . .
Puma_concolor                     . . . . . . . . . . . . . . . . . . . .
Puma_yagouaroundi                 . . . . . . . . . . . . . . . . . . . .
Lynx_canadensis                   . . . . . . . . . . . . . . . . . . . .
Lynx_pardinus                     . . . . . . . . . . . . . . . . . . . .
Lynx_rufus                        . . . . . . . . . . . . . . . . . . . .
Leopardus_geoffroyi               . . . . . . . . . . . . . . A . . . . .
Caracal_caracal                   . . . . . . . . . . . . . . . . . . . .
Neofelis_diardi                   . . . . . . . . . . . . . . . . . . . .
Neofelis_nebulosa                 . . . . . . . . . . . . . . . . . . . .
Panthera_leo                      . . . . . . . . . . . . . . . . . . . .
Panthera_onca                     . . . . . . . . . . . . . . . . . . . .
Panthera_pardus                   . . . . . . . . . . . . . . . . . . . .
Panthera_pardus_nimr              . . . . . . . . . . . . . . . . . . . .
Panthera_tigris_altaica           . . . . . . . . . . . . . . . . . . . .
Panthera_tigris_jacksoni          . . . . . . . . . . . . . . . . . . . .
Panthera_tigris_tigris            . . . . . . . . . . . . . . . . . . . .
Panthera_uncia                    . . . . . . . . . . . . . . . . . . . .
Crocuta_crocuta                   . . . . . . . . . . . . . . . . . . . .
Hyaena_hyaena                     . . . . . . . . . . . . . . . . . . . .
Proteles_cristata_cristata        . . . . . . . . . . . . . . . . . . . .
Canis_lupus_familiaris            . . . . . . . . . . . . . . A . . . . .
Ailuropoda_melanoleuca            . . . . . . . . . . . . . . A . . . . .
Paguma_larvata                    . . . . . . . . . . . . . . . . . . . .
Paradoxurus_hermaphroditus        . . . . . . . . . . . . . . . . . . . .
Neovison_vison                    . . . . . . . . . . . . . . A . . . . .
Potos_flavus                      . . . . . . . . . . . . . . A . . . . .
Desmodus_rotundus                 . . . . . G . . . . . C . . T . . . . .
Sarcophilus_harrisii              . . G . . G . . C . . . . . A . . . . .
Sminthopsis_crassicaudata         . . G . . G . . . . . . . . . . . . . .
Phascolarctos_cinereus            . . G . . G . . . . . . . . . . . . . .
Erinaceus_europaeus               . . . . . T . . . . . . . . C . . . . .
Talpa_europaea                    . . . . . G . . . . . . . . C . . . . .
Ornithorhynchus_anatinus          . . G . . G . . C . . C . . C . . . . .
Tachyglossus_aculeatus            . . . . . G . . C . . . . . C . . . . .
Macaca_fascicularis               . . . . . G . . . . . . . . C . . . . .
```

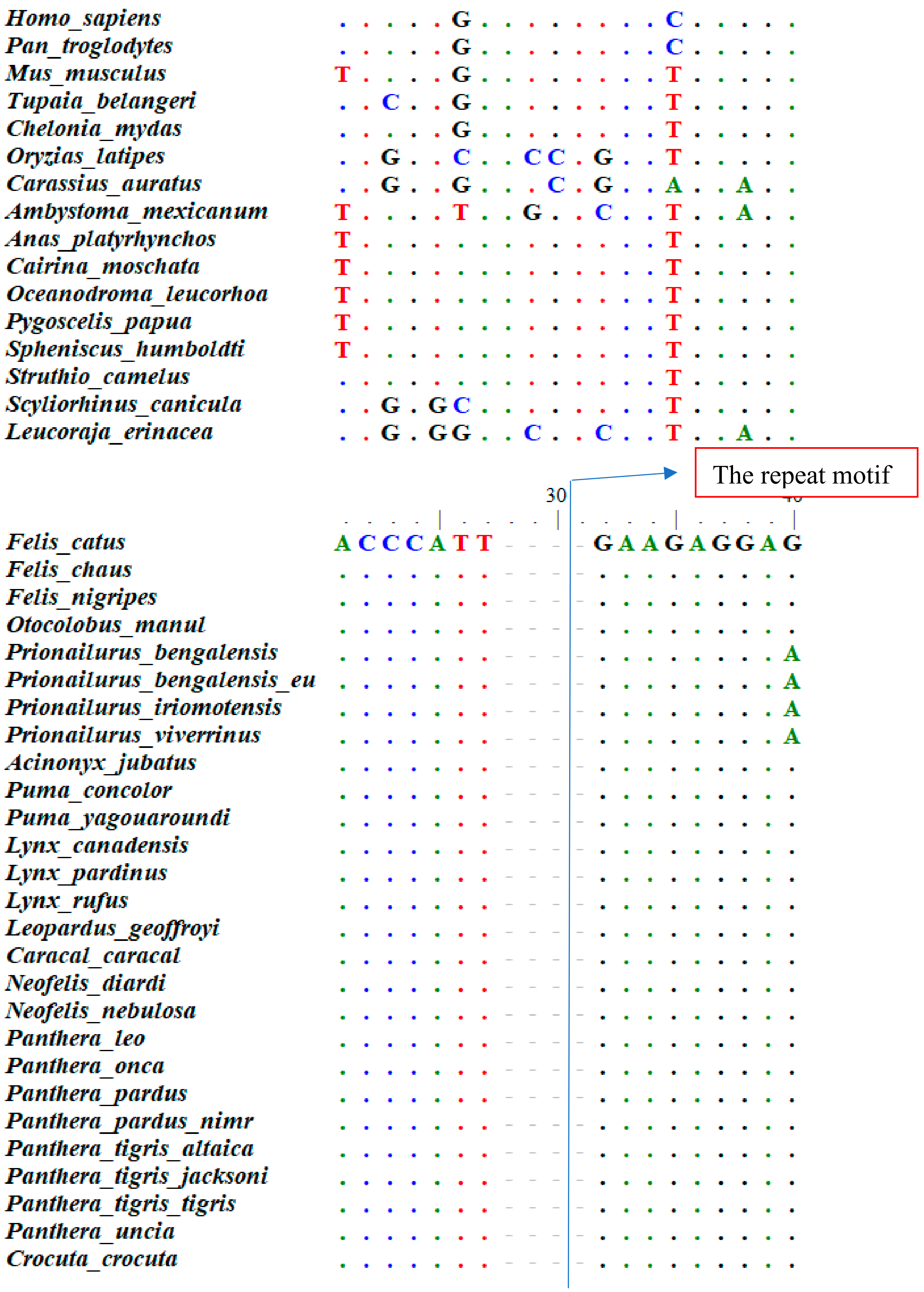
Homo_sapiens
Pan_troglodytes
Mus_musculus
Tupaia_belangeri
Chelonia_mydas
Oryzias_latipes
Carassius_auratus
Ambystoma_mexicanum
Anas_platyrhynchos
Cairina_moschata
Oceanodroma_leucorhoa
Pygoscelis_papua
Spheniscus_humboldti
Struthio_camelus
Scyliorhinus_canicula
Leucoraja_erinacea
The repeat motif
30
Felis_catus
ACCCATT GAAGAGGAG
Felis_chaus
Felis_nigripes
Otocolobus_manul
Prionailurus_bengalensis
Prionailurus_bengalensis_eu
Prionailurus_iriomotensis
Prionailurus_viverrinus
Acinonyx_jubatus
Puma_concolor
Puma_yagouaroundi
Lynx_canadensis
Lynx_pardinus
Lynx_rufus
Leopardus_geoffroyi
Caracal_caracal
Neofelis_diardi
Neofelis_nebulosa
Panthera_leo
Panthera_onca
Panthera_pardus
Panthera_pardus_nimr
Panthera_tigris_altaica
Panthera_tigris_jacksoni
Panthera_tigris_tigris
Panthera_uncia
Crocuta_crocuta

Hyaena_hyaena	`.......---|-.........`
Proteles_cristata_cristata	`.......---|-.........`
Canis_lupus_familiaris	`.......---|-........A`
Ailuropoda_melanoleuca	`.......---|-..G.....A`
Paguma_larvata	`.......---|-.........`
Paradoxurus_hermaphroditus	`.......---|-.........`
Neovison_vison	`.......---|-........A`
Potos_flavus	`.......---|-........A`
Desmodus_rotundus	`.......---|-.........`
Sarcophilus_harrisii	`G..T...---|-..G.....A`
Sminthopsis_crassicaudata	`G..T...---|-..G..A..A`
Phascolarctos_cinereus	`G..T...---|-..G......`
Erinaceus_europaeus	`G..T...---|-.........`
Talpa_europaea	`.......---|-.........`
Ornithorhynchus_anatinus	`G..T...---|-..G......`
Tachyglossus_aculeatus	`G..T...---|-.........`
Macaca_fascicularis	`.......---|-.........`
Homo_sapiens	`.......---|-.........`
Pan_troglodytes	`.......---|-.........`
Mus_musculus	`.......---|-..G..A...`
Tupaia_belangeri	`.......---|-.........`
Chelonia_mydas	`G..T...---|-..G..A..A`
Oryzias_latipes	`G.....CAAA|----......`
Carassius_auratus	`G..T...---|-.....A...`
Ambystoma_mexicanum	`...A..C---|-..G..A...`
Anas_platyrhynchos	`G..T...---|-..G.....A`
Cairina_moschata	`G..T...---|-..G......`
Oceanodroma_leucorhoa	`G..T...---|-..G.....A`
Pygoscelis_papua	`G..T...---|-..G.....A`
Spheniscus_humboldti	`G..T...---|-..G.....A`
Struthio_camelus	`G..T...---|-..G......`
Scyliorhinus_canicula	`G..G..CCAG|----......`
Leucoraja_erinacea	`......CCAG|----......`

	50 60
Felis_catus	`GAAGAGGAGGAGGAGGAGGA`
Felis_chaus	`....................`
Felis_nigripes	`....................`
Otocolobus_manul	`....................`
Prionailurus_bengalensis	`....................`
Prionailurus_bengalensis_eu	`....................`
Prionailurus_iriomotensis	`....................`
Prionailurus_viverrinus	`....................`
Acinonyx_jubatus	`....................`
Puma_concolor	`....................`

Puma_yagouaroundi	. . . .
Lynx_canadensis	. . . .
Lynx_pardinus	. . . .
Lynx_rufus	. . . .
Leopardus_geoffroyi	. . . .
Caracal_caracal	. . . .
Neofelis_diardi	. . . . T . . .
Neofelis_nebulosa	. . . . A . .
Panthera_leo	. . . .
Panthera_onca	. . . .
Panthera_pardus	. . . .
Panthera_pardus_nimr	. . . .
Panthera_tigris_altaica	. . . .
Panthera_tigris_jacksoni	. . . .
Panthera_tigris_tigris	. . . .
Panthera_uncia	. . . .
Crocuta_crocuta	. . . . A . .
Hyaena_hyaena	. . . . A . .
Proteles_cristata_cristata	. . . . A . .
Canis_lupus_familiaris	. . G . . A . . A . . . .
Ailuropoda_melanoleuca	. . . . A . . . . .
Paguma_larvata	. . . . . A . . . . A . . . . .
Paradoxurus_hermaphroditus	. . . . . A . . . .
Neovison_vison	. . . . A . . A . . . . .
Potos_flavus	. . . . A . . . . . A . . . . .
Desmodus_rotundus	. . G . . A . . . .
Sarcophilus_harrisii	. . G . . . . A . .
Sminthopsis_crassicaudata	. . G . . . .
Phascolarctos_cinereus	. . . . A . .
Erinaceus_europaeus	. . . . A . . . .
Talpa_europaea	. . . . A . . A . . . . .
Ornithorhynchus_anatinus	. . . . . A . . . . A . .
Tachyglossus_aculeatus	. . G . . A . . . . A . .
Macaca_fascicularis	. . . . A . .
Homo_sapiens	. . . .
Pan_troglodytes	. . . .
Mus_musculus	. . G . . . . A . .
Tupaia_belangeri	. . . . . A . . . . A . .
Chelonia_mydas	. . . . A - -
Oryzias_latipes	. . G . . . . A . . . .
Carassius_auratus	. . G . . A . . . . ~ ~ ~ ~ ~
Ambystoma_mexicanum	. . . . T . . T . . C . . . - -
Anas_platyrhynchos	. . G . . . . . A . . A . . A - - - - -
Cairina_moschata	. . G . . . . . R . . A . . A - - - - -
Oceanodroma_leucorhoa	. . . . A . . A - - - - -
Pygoscelis_papua	. . . . A - - - - -

```
Spheniscus_humboldti              . . . . . . . . . . . . . . A - - - - -
Struthio_camelus                  . . . . . A . . . . . A . . . - - - - -
Scyliorhinus_canicula             . . T . . . . . C . . . - - - - - - - -
Leucoraja_erinacea                . . G . . . . . C . . . . . C . . . - -

                                                      70                  80
                                  . . . . | . . . . | . . . . | . . . . |
Felis_catus                       G G A G G A A - - - - - - - - - - - - -
Felis_chaus                       . . . . . . G G A A - - - - - - - - - -
Felis_nigripes                    . . . . . . . - - - - - - - - - - - - -
Otocolobus_manul                  . . . . . . . - - - - - - - - - - - - -
Prionailurus_bengalensis          . . . . . . G G A A - - - - - - - - - -
Prionailurus_bengalensis_eu       . . . . . . G G A A - - - - - - - - - -
Prionailurus_iriomotensis         . . . . . . G G A A - - - - - - - - - -
Prionailurus_viverrinus           . . . . . . G G A A - - - - - - - - - -
Acinonyx_jubatus                  . . . . . . . - - - - - - - - - - - - -
Puma_concolor                     . . . . . . . - - - - - - - - - - - - -
Puma_yagouaroundi                 . . . . . . . - - - - - - - - - - - - -
Lynx_canadensis                   . . . . . . . - - - - - - - - - - - - -
Lynx_pardinus                     . . . . . . . - - - - - - - - - - - - -
Lynx_rufus                        . . . . . . G G A A - - - - - - - - - -
Leopardus_geoffroyi               A . . . . . . - - - - - - - - - - - - -
Caracal_caracal                   . . . . . . . - - - - - - - - - - - - -
Neofelis_diardi                   . . . A - - - - - - - - - - - - - - - -
Neofelis_nebulosa                 . . . A - - - - - - - - - - - - - - - -
Panthera_leo                      . . . . . . . - - - - - - - - - - - - -
Panthera_onca                     . . . . . . . - - - - - - - - - - - - -
Panthera_pardus                   . . . . . . . - - - - - - - - - - - - -
Panthera_pardus_nimr              . . . . . . . - - - - - - - - - - - - -
Panthera_tigris_altaica           . . . . . . . - - - - - - - - - - - - -
Panthera_tigris_jacksoni          . . . . . . . - - - - - - - - - - - - -
Panthera_tigris_tigris            A - - - - - - - - - - - - - - - - - - -
Panthera_uncia                    . . . . . . . - - - - - - - - - - - - -
Crocuta_crocuta                   . . . A - - - - - - - - - - - - - - - -
Hyaena_hyaena                     . . . A - - - - - - - - - - - - - - - -
Proteles_cristata_cristata        . . . A - - - - - - - - - - - - - - - -
Canis_lupus_familiaris            . - - - - - - - - - - - - - - - - - - -
Ailuropoda_melanoleuca            . - - - - - - - - - - - - - - - - - - -
Paguma_larvata                    . . . . . . . - - - - - - - - - - - - -
Paradoxurus_hermaphroditus        . . . . . . . - - - - - - - - - - - - -
Neovison_vison                    A - - - - - - - - - - - - - - - - - - -
Potos_flavus                      . - - - - - - - - - - - - - - - - - - -
Desmodus_rotundus                 A - - - - - - - - - - - - - - - - - - -
Sarcophilus_harrisii              . . . . . . G - - - - - - - - - - - - -
Sminthopsis_crassicaudata         A . . . . . . - - - - - - - - - - - - -
Phascolarctos_cinereus            T . . A . . G G A G - - - - - - - - - -
```

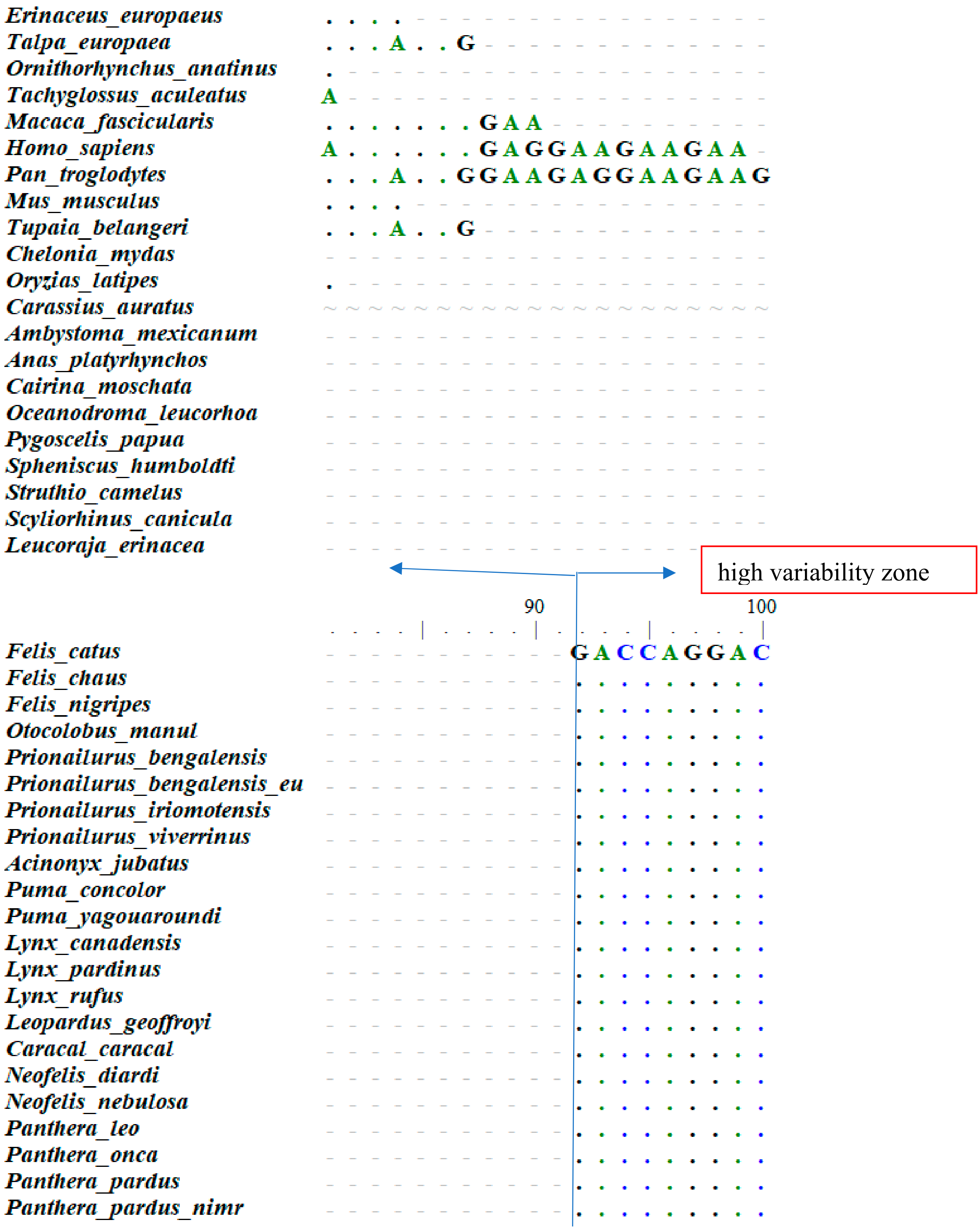

Erinaceus_europaeus
Talpa_europaea . . . A . . G
Ornithorhynchus_anatinus
Tachyglossus_aculeatus A
Macaca_fascicularis . . . . GAA
Homo_sapiens A . . . . GAGGAAGAAGAA
Pan_troglodytes . . . A . . GGAAGAGGAAGAAG
Mus_musculus
Tupaia_belangeri . . . A . . G
Chelonia_mydas
Oryzias_latipes
Carassius_auratus
Ambystoma_mexicanum
Anas_platyrhynchos
Cairina_moschata
Oceanodroma_leucorhoa
Pygoscelis_papua
Spheniscus_humboldti
Struthio_camelus
Scyliorhinus_canicula
Leucoraja_erinacea
high variability zone
90
100
Felis_catus GACCAGGAC
Felis_chaus
Felis_nigripes
Otocolobus_manul
Prionailurus_bengalensis
Prionailurus_bengalensis_eu
Prionailurus_iriomotensis
Prionailurus_viverrinus
Acinonyx_jubatus
Puma_concolor
Puma_yagouaroundi
Lynx_canadensis
Lynx_pardinus
Lynx_rufus
Leopardus_geoffroyi
Caracal_caracal
Neofelis_diardi
Neofelis_nebulosa
Panthera_leo
Panthera_onca
Panthera_pardus
Panthera_pardus_nimr

```
Panthera_tigris_altaica           ----------- | .........
Panthera_tigris_jacksoni          ----------- | .........
Panthera_tigris_tigris            ----------- | .........
Panthera_uncia                    ----------- | .........
Crocuta_crocuta                   ----------- | .....A...
Hyaena_hyaena                     ----------- | .....A...
Proteles_cristata_cristata        ----------- | .....A...
Canis_lupus_familiaris            ----------- | ..T......
Ailuropoda_melanoleuca            ----------- | .........
Paguma_larvata                    ----------- | .........
Paradoxurus_hermaphroditus        ----------- | .........
Neovison_vison                    ----------- | .........
Potos_flavus                      ----------- | .........
Desmodus_rotundus                 ----------- | .........
Sarcophilus_harrisii              ----------- | .........
Sminthopsis_crassicaudata         ----------- | .........
Phascolarctos_cinereus            ----------- | .........
Erinaceus_europaeus               ----------- | .........
Talpa_europaea                    ----------- | .........
Ornithorhynchus_anatinus          ----------- | ---.....T
Tachyglossus_aculeatus            ----------- | ---.....T
Macaca_fascicularis               ----------- | .........
Homo_sapiens                      ----------- | .........
Pan_troglodytes                   AA--------- | .C.......
Mus_musculus                      ----------- | .........
Tupaia_belangeri                  --------GTG | .........
Chelonia_mydas                    ----------- | ..TG....T
Oryzias_latipes                   --GACGCCAGT | ...G.....
Carassius_auratus                 ~~GAT---GGA | ..GG.A...
Ambystoma_mexicanum               ----------- | ..TG....T
Anas_platyrhynchos                ----------- | ..T.....T
Cairina_moschata                  ----------- | ..T.....T
Oceanodroma_leucorhoa             ----------- | ..T.....T
Pygoscelis_papua                  ----------- | ..T...T.T
Spheniscus_humboldti              ----------- | ..T...T..
Struthio_camelus                  ----------- | ..T.....T
Scyliorhinus_canicula             --GATGATGCA | ..G..A..T
Leucoraja_erinacea                --------ACG | ..G......

                                          110       120
                                  ....|....|....|....|
Felis_catus                       ATGGATGCAGATGACAGAGT
Felis_chaus                       ....................
Felis_nigripes                    ....................
Otocolobus_manul                  ....................
Prionailurus_bengalensis          ....................
```

Prionailurus_bengalensis_eu	. . . .
Prionailurus_iriomotensis	. . . .
Prionailurus_viverrinus	. . . .
Acinonyx_jubatus	. . . .
Puma_concolor	. . . .
Puma_yagouaroundi	. . . .
Lynx_canadensis	. . . .
Lynx_pardinus	. . . .
Lynx_rufus	. . . .
Leopardus_geoffroyi	. . . .
Caracal_caracal	. . . .
Neofelis_diardi	. . . .
Neofelis_nebulosa	. . . .
Panthera_leo	. . . .
Panthera_onca	. . . .
Panthera_pardus	. . . .
Panthera_pardus_nimr	. . . .
Panthera_tigris_altaica	. . . .
Panthera_tigris_jacksoni	. . . .
Panthera_tigris_tigris	. . . .
Panthera_uncia	. . . .
Crocuta_crocuta	. . . .
Hyaena_hyaena	. . . .
Proteles_cristata_cristata	. . . .
Canis_lupus_familiaris	. . . . G . .
Ailuropoda_melanoleuca	. . . . G . .
Paguma_larvata	. . . .
Paradoxurus_hermaphroditus	. . . .
Neovison_vison	. . . . G . .
Potos_flavus	. . . . G . .
Desmodus_rotundus	. . . . G . .
Sarcophilus_harrisii	. . . . A . . . . G A .
Sminthopsis_crassicaudata	. . . . A . . . . G A .
Phascolarctos_cinereus	. . . . A . . . . G A .
Erinaceus_europaeus	. . . . G . .
Talpa_europaea	. . . . G . .
Ornithorhynchus_anatinus	. . . . A . . . C . . G C . G . .
Tachyglossus_aculeatus	. . . . A . . . C . . G C . G . .
Macaca_fascicularis	. . . .
Homo_sapiens	. . . .
Pan_troglodytes	. . . .
Mus_musculus	. . . . C . . . .
Tupaia_belangeri	. . . . G . .
Chelonia_mydas	. . . . A G . . . . G . .
Oryzias_latipes	. . . . . C T . T . . . . G - -
Carassius_auratus	. . . . T . . C . . T . . . - -

```
Ambystoma_mexicanum              .......A............
Anas_platyrhynchos               .......A............
Cairina_moschata                 .......A............
Oceanodroma_leucorhoa            .......A............
Pygoscelis_papua                 .......A............
Spheniscus_humboldti             .......A............
Struthio_camelus                 .......A......T.....
Scyliorhinus_canicula            .....G.........C.G..
Leucoraja_erinacea               .....G.....C...C.T..

                                          130       140
                                 ....|....|....|....|
Felis_catus                      GGTGGTAGAGTACCAT----
Felis_chaus                      ...............C----
Felis_nigripes                   ...............C----
Otocolobus_manul                 ...............C----
Prionailurus_bengalensis         .........A.....C----
Prionailurus_bengalensis_eu      .........A.....C----
Prionailurus_iriomotensis        .........A.....C----
Prionailurus_viverrinus          .........A.....C----
Acinonyx_jubatus                 ...............C----
Puma_concolor                    ...............C----
Puma_yagouaroundi                ...............C----
Lynx_canadensis                  ...............C----
Lynx_pardinus                    ...............C----
Lynx_rufus                       ...............C----
Leopardus_geoffroyi              ...............C----
Caracal_caracal                  ...............C----
Neofelis_diardi                  ...............C----
Neofelis_nebulosa                ...............C----
Panthera_leo                     ...............C----
Panthera_onca                    ...............C----
Panthera_pardus                  ...............C----
Panthera_pardus_nimr             ...............C----
Panthera_tigris_altaica          ...............C----
Panthera_tigris_jacksoni         ...............C----
Panthera_tigris_tigris           ...............C----
Panthera_uncia                   ...............C----
Crocuta_crocuta                  .......T........----
Hyaena_hyaena                    .......T........----
Proteles_cristata_cristata       .......T........----
Canis_lupus_familiaris           ...A............----
Ailuropoda_melanoleuca           .......G.......C----
Paguma_larvata                   ...............C----
Paradoxurus_hermaphroditus       ...............C----
Neovison_vison                   .......G........----
```

```
Potos_flavus                    . . . . . . . . . . . . . . . . - - - -
Desmodus_rotundus               . . . . . . G . . . . . . . . . - - - -
Sarcophilus_harrisii            C . . . . . G . . A . . . . G G - - - -
Sminthopsis_crassicaudata       T . . . . . G . . A . . . . G G - - - -
Phascolarctos_cinereus          C . . A . . G . . . . . . . G G - - - -
Erinaceus_europaeus             . . . . . . . . . . . . . . . . - - - -
Talpa_europaea                  . . . . . . C . . . . . . . . . - - - -
Ornithorhynchus_anatinus        . . . . . . T . . . . . . . G G - - - -
Tachyglossus_aculeatus          . . . . . . T . . . . . . . G G - - - -
Macaca_fascicularis             . . . . . . . . . . . . . . . C - - - -
Homo_sapiens                    . . . . . . . . . . . . . . . C - - - -
Pan_troglodytes                 . . . . . . . . . . . . . . . C - - - -
Mus_musculus                    C . . . . . G . . . . . . . . . - - - -
Tupaia_belangeri                . . . . . . . . . . . . . . . . - - - -
Chelonia_mydas                  T . . . . . . . . . . . . . G G - - - -
Oryzias_latipes                 - . . . . . G . . . . T T A . G - - - -
Carassius_auratus               - . . A . . G . . . . . T A . A G A T C
Ambystoma_mexicanum             T . . . . . G . . A . . . . . . - - - -
Anas_platyrhynchos              T . . T . . . . . A . . . . G . - - - -
Cairina_moschata                T . . T . . . . . A . . . . G . - - - -
Oceanodroma_leucorhoa           T . . . . . . . . . . . . . G . - - - -
Pygoscelis_papua                T . . . . . . . . . . . . . G . - - - -
Spheniscus_humboldti            T . . . . . . . . . . . . . G . - - - -
Struthio_camelus                T . . . . . T . . . . . . . G C - - - -
Scyliorhinus_canicula           . . . . . . T . . A . . T . G C - - - -
Leucoraja_erinacea              . . . . . . G . . . . . T . G C - - - -

                                                   150                 160
                                . . . . | . . . . | . . . . | . . . . |
Felis_catus                     - - - - - - - - - - - G A G G A G C T C
Felis_chaus                     - - - - - - - - - - - . . . . . . . . .
Felis_nigripes                  - - - - - - - - - - - . . . . . . . . .
Otocolobus_manul                - - - - - - - - - - - . . . . . . . . .
Prionailurus_bengalensis        - - - - - - - - - - - . . . . . . . . .
Prionailurus_bengalensis_eu     - - - - - - - - - - - . . . . . . . . .
Prionailurus_iriomotensis       - - - - - - - - - - - . . . . . . . . .
Prionailurus_viverrinus         - - - - - - - - - - - . . . . . . . . .
Acinonyx_jubatus                - - - - - - - - - - - . . . . . . . . .
Puma_concolor                   - - - - - - - - - - - . . . . . . . . .
Puma_yagouaroundi               - - - - - - - - - - - . . . . . . . . .
Lynx_canadensis                 - - - - - - - - - - - . . . . . . . . .
Lynx_pardinus                   - - - - - - - - - - - . . . . . . . . .
Lynx_rufus                      - - - - - - - - - - - . . . . . . . . .
Leopardus_geoffroyi             - - - - - - - - - - - . . . . . . . . .
Caracal_caracal                 - - - - - - - - - - - . . . . . . . . .
Neofelis_diardi                 - - - - - - - - - - - . . . . . . . . .
```

Neofelis_nebulosa	-----------.........
Panthera_leo	-----------.........
Panthera_onca	-----------.........
Panthera_pardus	-----------.........
Panthera_pardus_nimr	-----------.........
Panthera_tigris_altaica	-----------.........
Panthera_tigris_jacksoni	-----------.........
Panthera_tigris_tigris	-----------.........
Panthera_uncia	-----------.........
Crocuta_crocuta	-----------........T
Hyaena_hyaena	-----------........T
Proteles_cristata_cristata	-----------........T
Canis_lupus_familiaris	-----------.........
Ailuropoda_melanoleuca	-----------........T
Paguma_larvata	-----------.........
Paradoxurus_hermaphroditus	-----------.........
Neovison_vison	-----------.........
Potos_flavus	-----------.........
Desmodus_rotundus	-----------.........
Sarcophilus_harrisii	-----------..T.....G
Sminthopsis_crassicaudata	-----------..C.....G
Phascolarctos_cinereus	-----------..T.....G
Erinaceus_europaeus	-----------.....A...
Talpa_europaea	-----------.........
Ornithorhynchus_anatinus	-----------..C.....G
Tachyglossus_aculeatus	-----------..C.....G
Macaca_fascicularis	-----------.........
Homo_sapiens	-----------.........
Pan_troglodytes	-----------.........
Mus_musculus	-----------.........
Tupaia_belangeri	-----------.........
Chelonia_mydas	-----------..T..A..A
Oryzias_latipes	-----------..CA.AGGA
Carassius_auratus	GGGGAGACCGT..ACGAGGG
Ambystoma_mexicanum	-----------..T..A...
Anas_platyrhynchos	-----------..T..A..G
Cairina_moschata	-----------..T..A..G
Oceanodroma_leucorhoa	-----------..T..A..G
Pygoscelis_papua	-----------..T..A..G
Spheniscus_humboldti	-----------..T..A..G
Struthio_camelus	-----------..T..A..G
Scyliorhinus_canicula	-----------A.C...AGT
Leucoraja_erinacea	-----------A.C...AG.

170 180

Felis_catus	C C A G C T C T C A A G C A G A C C C G
Felis_chaus	. . . .
Felis_nigripes	. . . .
Otocolobus_manul	. . . .
Prionailurus_bengalensis	. . . .
Prionailurus_bengalensis_eu	. . . .
Prionailurus_iriomotensis	. . . .
Prionailurus_viverrinus	. . . .
Acinonyx_jubatus	. . . .
Puma_concolor	. . . .
Puma_yagouaroundi	. . . .
Lynx_canadensis	. . . .
Lynx_pardinus	. . . .
Lynx_rufus	. . . .
Leopardus_geoffroyi	. . . .
Caracal_caracal	. . . .
Neofelis_diardi	. . . .
Neofelis_nebulosa	. . . .
Panthera_leo	. . . .
Panthera_onca	. . . .
Panthera_pardus	. . . .
Panthera_pardus_nimr	. . . .
Panthera_tigris_altaica	. . . .
Panthera_tigris_jacksoni	. . . .
Panthera_tigris_tigris	. . . .
Panthera_uncia	. . . .
Crocuta_crocuta	. . . .
Hyaena_hyaena	. . . .
Proteles_cristata_cristata	. . . .
Canis_lupus_familiaris	. . . .
Ailuropoda_melanoleuca	. . . . A . C A . . . . .
Paguma_larvata	. . . . . . G . . . .
Paradoxurus_hermaphroditus	. . . . . . G . . . .
Neovison_vison	. . . .
Potos_flavus	. . . .
Desmodus_rotundus	. . . .
Sarcophilus_harrisii	. A G . . C T . . . . A G . G . .
Sminthopsis_crassicaudata	. A G . . C T . . . . A G . G . .
Phascolarctos_cinereus	. A . . . C T . . . . G . G . .
Erinaceus_europaeus	. . . . G . . . . A .
Talpa_europaea	. . C A . A . . . .
Ornithorhynchus_anatinus	. A . . . . T . . . G . . . . G . T . .
Tachyglossus_aculeatus	. A G . . . T . . . G . . . . G . T . .
Macaca_fascicularis	. . G . . . . C . C . . . .
Homo_sapiens	. . G . . . . C . . . .
Pan_troglodytes	. . G . . . . C . . . .

```
Mus_musculus                    ....G..........C....
Tupaia_belangeri                ........T...........
Chelonia_mydas                  .A....T....A..AT.T..
Oryzias_latipes                 GGCAGCTCACGTTCTGGAG.
Carassius_auratus               ...CGG..TGG.G.AGGAGA
Ambystoma_mexicanum             .A....T.T..A..AT.T..
Anas_platyrhynchos              .A.C..T....A...T.T..
Cairina_moschata                .A.C..T....A...T.T..
Oceanodroma_leucorhoa           .A....T....A...T....
Pygoscelis_papua                .AG...T....A...T....
Spheniscus_humboldti            .AG...T....A...T....
Struthio_camelus                .A....T....A...T.T..
Scyliorhinus_canicula           --------------------
Leucoraja_erinacea              --------------------
```

```
                                          190       200
                                ....|....|....|....|
Felis_catus                     GGAGCGGAGCACATCTAGGC
Felis_chaus                     ....................
Felis_nigripes                  ....................
Otocolobus_manul                ....................
Prionailurus_bengalensis        ....................
Prionailurus_bengalensis_eu     ....................
Prionailurus_iriomotensis       ....................
Prionailurus_viverrinus         ....................
Acinonyx_jubatus                ....................
Puma_concolor                   ....................
Puma_yagouaroundi               ....................
Lynx_canadensis                 ....................
Lynx_pardinus                   ....................
Lynx_rufus                      ....................
Leopardus_geoffroyi             ....................
Caracal_caracal                 ....................
Neofelis_diardi                 ...............G....
Neofelis_nebulosa               ...............G....
Panthera_leo                    ...............G....
Panthera_onca                   ...............G....
Panthera_pardus                 ...............G....
Panthera_pardus_nimr            ...............G....
Panthera_tigris_altaica         ...............G....
Panthera_tigris_jacksoni        ...............G....
Panthera_tigris_tigris          ...............G....
Panthera_uncia                  ...............G....
Crocuta_crocuta                 ....................
Hyaena_hyaena                   ....................
Proteles_cristata_cristata      ....................
```

```
Canis_lupus_familiaris            . . . . . . . . . . . . . . . . . . . .
Ailuropoda_melanoleuca            . . . . . . . . . . . . . . . . . . . .
Paguma_larvata                    . . . . . . . . . . . . . . . C . . . .
Paradoxurus_hermaphroditus        . . . . . . . . . . . . . . . . . . . .
Neovison_vison                    . . . A . . . . . . . . . . . . . . . .
Potos_flavus                      . . . . . . . . . . . . . . . . . . . .
Desmodus_rotundus                 . . . . . . . . . . G . . . . C . . . .
Sarcophilus_harrisii              C . . A . . A . . . G G C . . C C . C .
Sminthopsis_crassicaudata         C . . A . . A . . . G G C . . C C . C .
Phascolarctos_cinereus            C . . . . . A . . T G . . . . C C . T .
Erinaceus_europaeus               . . . . A . . . . T G . . . . A . . . A
Talpa_europaea                    . . . A . . . . . T G . T . . . . . . .
Ornithorhynchus_anatinus          . . . C . . C . . T G G C . . G . . . A
Tachyglossus_aculeatus            . . . C . . C . . T G G C . . G . . . A
Macaca_fascicularis               . . . . . . . . . T G . G . . . . . A .
Homo_sapiens                      . . . . . . . . . . G . G . . . . . A .
Pan_troglodytes                   . . . . . . . . . . G . G . . . . . A .
Mus_musculus                      T . . . . . . . . . C T G . . . . . . .
Tupaia_belangeri                  A . . . . . . . . . G . G . . . . . . .
Chelonia_mydas                    A . . T . . C . . T G . . G . A . . A .
Oryzias_latipes                   . . C . G . . C T T C G C A G C . . T G
Carassius_auratus                 C C T . . . T . . T C G C . T A G . A G
Ambystoma_mexicanum               . . . . . A C . . . . G G G . . C . . .
Anas_platyrhynchos                A . . C . . C . . T G . . G . A . . A .
Cairina_moschata                  A . . C . . C . . Y G . . G . A . . A .
Oceanodroma_leucorhoa             A . . C . . T . . T G . . G . A . . A .
Pygoscelis_papua                  A . . C . . C . . T G . . G . A . . A .
Spheniscus_humboldti              A . . C . . C . . T G . . G . A . . A .
Struthio_camelus                  A . . C . . C . . T G . . G . C . . A .
Scyliorhinus_canicula             - - - - . . C . C G G G T G G G C A C T
Leucoraja_erinacea                - - - - . . . . C G G G G - - - C C C T

                                                    210                 220
                                  . . . . | . . . . | . . . . | . . . . |
Felis_catus                       A G T C C A G T G C C A G C A G T - - -
Felis_chaus                       . . . . . . . . . . . . . . . . . - - -
Felis_nigripes                    . . . . . . . . . . . . . . . . . - - -
Otocolobus_manul                  . . . . . . . . . . . . . . . . . - - -
Prionailurus_bengalensis          . . . . . . . . . . . . . . . . . - - -
Prionailurus_bengalensis_eu       . . . . . . . . . . . . . . . . . - - -
Prionailurus_iriomotensis         . . . . . . . . . . . . . . . . . - - -
Prionailurus_viverrinus           . . . . . . . . . . . . . . . . . - - -
Acinonyx_jubatus                  . . . . . . . . . . . . . . . . . - - -
Puma_concolor                     . . . . . . . . . . . . . . . . . - - -
Puma_yagouaroundi                 . . . . . . . . . . . . . . . . . - - -
Lynx_canadensis                   . . . . . . . . . . . . . . . . . - - -
```

Lynx_pardinus	`.................---`
Lynx_rufus	`.................---`
Leopardus_geoffroyi	`.................---`
Caracal_caracal	`.................---`
Neofelis_diardi	`.................---`
Neofelis_nebulosa	`.................---`
Panthera_leo	`..........A......---`
Panthera_onca	`.................---`
Panthera_pardus	`.................---`
Panthera_pardus_nimr	`..........A......---`
Panthera_tigris_altaica	`.................---`
Panthera_tigris_jacksoni	`.................---`
Panthera_tigris_tigris	`.................---`
Panthera_uncia	`.................---`
Crocuta_crocuta	`.................---`
Hyaena_hyaena	`.................---`
Proteles_cristata_cristata	`.................---`
Canis_lupus_familiaris	`.................---`
Ailuropoda_melanoleuca	`.................---`
Paguma_larvata	`.................---`
Paradoxurus_hermaphroditus	`.................---`
Neovison_vison	`.......C.........---`
Potos_flavus	`.................---`
Desmodus_rotundus	`G...............C---`
Sarcophilus_harrisii	`GCG....C......G.CTCG`
Sminthopsis_crassicaudata	`GCC....C......G.CTCG`
Phascolarctos_cinereus	`G.C....C..T...G..TCG`
Erinaceus_europaeus	`G.........T.....C---`
Talpa_europaea	`G.C.............C---`
Ornithorhynchus_anatinus	`G............TGAC---`
Tachyglossus_aculeatus	`G......C.....TGAC---`
Macaca_fascicularis	`GA..............C---`
Homo_sapiens	`GA..............C---`
Pan_troglodytes	`G...............C---`
Mus_musculus	`GA........G.....C---`
Tupaia_belangeri	`G...............C---`
Chelonia_mydas	`GA..A........TGCG---`
Oryzias_latipes	`C.GAGC.AT.TCCGTC.CCG`
Carassius_auratus	`GCC.ATCAC.TGCATC.---`
Ambystoma_mexicanum	`GA..G..CA....T.CC---`
Anas_platyrhynchos	`GA..A.....A..TGCA---`
Cairina_moschata	`GA..A.....A..TGCA---`
Oceanodroma_leucorhoa	`G...A.....T..TGCA---`
Pygoscelis_papua	`GA..G.....T...GCA---`
Spheniscus_humboldti	`GA..G.....T...GCA---`
Struthio_camelus	`GA..A.....T..TGCA---`

```
Scyliorhinus_canicula            T T A G . C C C C G G G . T T C . - - -
Leucoraja_erinacea               C T A G . C C C C A . G C . T C A - - -

                                                   230                 240
                                 . . . . | . . . . | . . . . | . . . . |
Felis_catus                      - - - T C A G A C T C C G A T G A A A T
Felis_chaus                      - - - . . . . . . . . . . . . . . . . .
Felis_nigripes                   - - - . . . . . . . . . . . . . . . . .
Otocolobus_manul                 - - - . . . . . . . . T . . . . . . . .
Prionailurus_bengalensis         - - - . . . . . . . . . . . . . . . . .
Prionailurus_bengalensis_eu      - - - . . . . . . . . . . . . . . . . .
Prionailurus_iriomotensis        - - - . . . . . . . . . . . . . . . . .
Prionailurus_viverrinus          - - - . . . . . . . . . . . . . . . . .
Acinonyx_jubatus                 - - - . . . . . . . . . . . . . . . . .
Puma_concolor                    - - - . . . . . . . . . . . . . . . . .
Puma_yagouaroundi                - - - . . . . . . . . . . . . . . . . .
Lynx_canadensis                  - - - . . . . . . . . . . . . . . . . .
Lynx_pardinus                    - - - . . . . . . . . . . . . . . . . .
Lynx_rufus                       - - - . . . . . . . . . . . . . . . . .
Leopardus_geoffroyi              - - - . . . . . . . . . . . . . . . . .
Caracal_caracal                  - - - . . . . . . . . . . . . . . . . .
Neofelis_diardi                  - - - . . . . . . . . . . . . . . . . .
Neofelis_nebulosa                - - - . . . . . . . . . . . . . . . . .
Panthera_leo                     - - - . . . . . . . . . . . . . . . . .
Panthera_onca                    - - - . . . . . . . . . . . . . . . . .
Panthera_pardus                  - - - . . . . . . . . . . . . . . . . .
Panthera_pardus_nimr             - - - . . . . . . . . . . . . . . . . .
Panthera_tigris_altaica          - - - . . . . . . . . . . . . . . . . .
Panthera_tigris_jacksoni         - - - . . . . . . . . . . . . . . . . .
Panthera_tigris_tigris           - - - . . . . . . . . . . . . . . . . .
Panthera_uncia                   - - - . . . . . . . . . . . . . . . . .
Crocuta_crocuta                  - - - . . . . . T . . T . . . . . . . .
Hyaena_hyaena                    - - - . . . . . T . . T . . . . . . . .
Proteles_cristata_cristata       - - - . . . . . T . . T . . . . . . . .
Canis_lupus_familiaris           - - - . . . . . . . . . . . . . . . . .
Ailuropoda_melanoleuca           - - - . . . . . . . . . . . C . . . . .
Paguma_larvata                   - - - . . . . . . . . T . . . . . . . .
Paradoxurus_hermaphroditus       - - - . . . . . . . . T . . . . . . . .
Neovison_vison                   - - - . . . . . . . . T . . . . . . . .
Potos_flavus                     - - - . . . . . . . . T . . . . . . . .
Desmodus_rotundus                - - - . . . . . . . . . . . . . . . . .
Sarcophilus_harrisii             G G C . . C . . . . . G . . C . . G . .
Sminthopsis_crassicaudata        G G C . . C . . . . . G . . C . . G . .
Phascolarctos_cinereus           G G C . . G . . . . . G . . C . . G . .
Erinaceus_europaeus              - - - . . C . . . . . T . . . . . . . .
Talpa_europaea                   - - - . . . . . . . . . . . C . . . . .
```

```
Ornithorhynchus_anatinus          ---........A.....G..
Tachyglossus_aculeatus            ---........A..C..G..
Macaca_fascicularis               ---........A........
Homo_sapiens                      ---........A........
Pan_troglodytes                   ---........A........
Mus_musculus                      ---........A.....G..
Tupaia_belangeri                  ---........A........
Chelonia_mydas                    ---..T..T..T........
Oryzias_latipes                   TGG.....G..G.....G..
Carassius_auratus                 ---.....T..A.....G..
Ambystoma_mexicanum               ---........T........
Anas_platyrhynchos                ---..T..T..T........
Cairina_moschata                  ---..T..T..T........
Oceanodroma_leucorhoa             ---..T..T..T........
Pygoscelis_papua                  ---..T..T..T........
Spheniscus_humboldti              ---..T..T..T........
Struthio_camelus                  ---.....T..T........
Scyliorhinus_canicula             ------.....T.....G..
Leucoraja_erinacea                ------.....T..C..G..

                                          250       260
                                  ....|....|....|....|
Felis_catus                       GGACTATGATCTAGAACTGA
Felis_chaus                       ....................
Felis_nigripes                    ....................
Otocolobus_manul                  ....................
Prionailurus_bengalensis          ....................
Prionailurus_bengalensis_eu       ....................
Prionailurus_iriomotensis         ....................
Prionailurus_viverrinus           ....................
Acinonyx_jubatus                  ....................
Puma_concolor                     ....................
Puma_yagouaroundi                 ....................
Lynx_canadensis                   ....................
Lynx_pardinus                     ....................
Lynx_rufus                        ....................
Leopardus_geoffroyi               ....................
Caracal_caracal                   ....................
Neofelis_diardi                   ....................
Neofelis_nebulosa                 ....................
Panthera_leo                      ....................
Panthera_onca                     ....................
Panthera_pardus                   ....................
Panthera_pardus_nimr              ....................
Panthera_tigris_altaica           ....................
Panthera_tigris_jacksoni          ....................
```

```
Panthera_tigris_tigris           ....................
Panthera_uncia                   ....................
Crocuta_crocuta                  ............G.......
Hyaena_hyaena                    ............G.......
Proteles_cristata_cristata       ............G.......
Canis_lupus_familiaris           ....................
Ailuropoda_melanoleuca           ....................
Paguma_larvata                   ..........T.......C.
Paradoxurus_hermaphroditus       ..........T.......C.
Neovison_vison                   ..................A.
Potos_flavus                     ...............G....
Desmodus_rotundus                ..........T.........
Sarcophilus_harrisii             .........C..G..G....
Sminthopsis_crassicaudata        ...T..C..C..G..G....
Phascolarctos_cinereus           ...T........G..GT...
Erinaceus_europaeus              ................T...
Talpa_europaea                   ....................
Ornithorhynchus_anatinus         ......C..CT.G.......
Tachyglossus_aculeatus           ......C..C..G.......
Macaca_fascicularis              ....................
Homo_sapiens                     ....................
Pan_troglodytes                  ....................
Mus_musculus                     ............G.......
Tupaia_belangeri                 ....................
Chelonia_mydas                   ............T.......
Oryzias_latipes                  ......C..C..G..G....
Carassius_auratus                ......C.....G.......
Ambystoma_mexicanum              ...T........C.......
Anas_platyrhynchos               ............T...T...
Cairina_moschata                 ............T...T...
Oceanodroma_leucorhoa            ............T.......
Pygoscelis_papua                 ............T.......
Spheniscus_humboldti             ............T.......
Struthio_camelus                 ............T.......
Scyliorhinus_canicula            ............G..GT...
Leucoraja_erinacea               ...T..C..C..G..G....

                                          270       280
                                 ....|....|....|....|
Felis_catus                      AAATGATTTCCACTCCTTCA
Felis_chaus                      ....................
Felis_nigripes                   ....................
Otocolobus_manul                 ....................
Prionailurus_bengalensis         ....................
Prionailurus_bengalensis_eu      ....................
Prionailurus_iriomotensis        ....................
```

```
Cairina_moschata                 . . . . . . . A . . A . . C . . C . . T
Oceanodroma_leucorhoa            . . . . . . . A . . A . . A . . C . . T
Pygoscelis_papua                 . . . . . . . A . . A . . A . . C . . T
Spheniscus_humboldti             . . . . . . . A . . A . . A . . C . . T
Struthio_camelus                 . . . . . . . A . . A . . C . . C . . T
Scyliorhinus_canicula            . G . . . . . C . . A . T G . . A . T G
Leucoraja_erinacea               . G . . . . . C . . . . T G . . C . . G

                                                   290                 300
                                 . . . . | . . . . | . . . . | . . . . |
Felis_catus                      C C A A A G A A A A G C A T G A A A A T
Felis_chaus                      . . . . . . . . . . . . . . . . . . . .
Felis_nigripes                   . . . . . . . . . . . . . . . . . . . .
Otocolobus_manul                 . . . . . . . . . . . . . . . . . . . .
Prionailurus_bengalensis         . . . . . . . . . . . . . . . . . . . .
Prionailurus_bengalensis_eu      . . . . . . . . . . . . . . . . . . . .
Prionailurus_iriomotensis        . . . . . . . . . . . . . . . . . . . .
Prionailurus_viverrinus          . . . . . . . . . . . . . . . . . . . .
Acinonyx_jubatus                 . . . . . . . . . . . . . . . . . . . .
Puma_concolor                    . . . . . . . . . . . . . . . . . . . .
Puma_yagouaroundi                . . . . . . . . . . . . . . . . . . . .
Lynx_canadensis                  . . . . . . . . . . . . . . . . . . . .
Lynx_pardinus                    . . . . . . . . . . . . . . . . . . . .
Lynx_rufus                       . . . . . . . . . . . . . . . . . . . .
Leopardus_geoffroyi              . . . . . . . . . . . . . . . . . . . .
Caracal_caracal                  . . . . . . . . . . . . . . . . . . . .
Neofelis_diardi                  . . . . . . . . . . . . . . . . . . . .
Neofelis_nebulosa                . . . . . . . . . . . . . . . . . . . .
Panthera_leo                     . . . . . . . . . . . . . . . . . . . .
Panthera_onca                    . . . . . . . . G . . . . . . . . . . .
Panthera_pardus                  . . . . . . . . . . . . . . . . . . . .
Panthera_pardus_nimr             . . . . . . . . . . . . . . . . . . . .
Panthera_tigris_altaica          . . . . . . . . . . . . . . . . . . . .
Panthera_tigris_jacksoni         . . . . . . . . . . . . . . . . . . . .
Panthera_tigris_tigris           . . . . . . . . . . . . . . . . . . . .
Panthera_uncia                   . . . . . . . . . . . . . . . . . . . .
Crocuta_crocuta                  . . . . . . . . . . . . . . . . . . . .
Hyaena_hyaena                    . . . . . . . . . . . . . . . . . . . .
Proteles_cristata_cristata       . . . . . . . . . . . . . . . . . . . .
Canis_lupus_familiaris           . . . . . . . . . . . . . . . . . . . .
Ailuropoda_melanoleuca           . . . . . . . . . . . . . . . . . . . .
Paguma_larvata                   . . . . . . . . . . . . . . . . . . . .
Paradoxurus_hermaphroditus       . . . . . . . . . . . . . . . . . . . .
Neovison_vison                   . . . . . . . . . . . . . . . . . . . .
Potos_flavus                     . . . . . . . . . . . . . . . . . . . .
Desmodus_rotundus                . . . . . . . . . . . . . . . . . . . .
```

```
Sarcophilus_harrisii              . . G . . . . . G . . . T . . . . G . .
Sminthopsis_crassicaudata         . . . . . . . . G . . . T . . . . G . .
Phascolarctos_cinereus            . . . . . . . . G . . . T . . . . G . .
Erinaceus_europaeus               . . . . . . . . . . . . . . . . . G . .
Talpa_europaea                    . . . . . . . . G . . . . . . . . . . .
Ornithorhynchus_anatinus          . . C . . A . . G . . . . . . . . G . .
Tachyglossus_aculeatus            . . C . . A . . G . . . . . . . . G . .
Macaca_fascicularis               . . . . . . . . . . . . . . . . . . . .
Homo_sapiens                      . . . . . . . . . . . . . . . . . . . .
Pan_troglodytes                   . . . . . . . . . . . . . . . . . . . .
Mus_musculus                      . . . . . . . . . . . . . . . . . . . .
Tupaia_belangeri                  . . . . . . . . . . . . . . . . . . . .
Chelonia_mydas                    . . C . . A . . G . . . . . . . . . . .
Oryzias_latipes                   . . . . . . . . . . . . . A A . . . . .
Carassius_auratus                 . . C . . A . . G . . . . . . . . G . .
Ambystoma_mexicanum               . . C . . A . . G . . T . . . . . G . .
Anas_platyrhynchos                . . C . . A . . G . . . . . . . . . . .
Cairina_moschata                  . . C . . A . . G . . . . . . . . . . .
Oceanodroma_leucorhoa             . . C . . A . . G . . . . . . . . . . .
Pygoscelis_papua                  . . C . . A . . G . . . . . . . . . . .
Spheniscus_humboldti              . . C . . A . . G . . . . . . . . . . .
Struthio_camelus                  . . C . . A . . . . . . . . . . . . . .
Scyliorhinus_canicula             . . C . . . . . G . . T C . . . . G . .
Leucoraja_erinacea                . . C . . . . . G . . T C . . . . G . .

                                                   310                 320
                                  . . . . | . . . . | . . . . | . . . . |
Felis_catus                       G A C C A T G T A C G C T G A T G A A G
Felis_chaus                       . . . . . . . . . . . . . . . . . . . .
Felis_nigripes                    . . . . . . . . . . . . . . . . . . . .
Otocolobus_manul                  . . . . . . . . . . . . . . . . . . . .
Prionailurus_bengalensis          . . . . . . . . . . . . . . . . . . . .
Prionailurus_bengalensis_eu       . . . . . . . . . . . . . . . . . . . .
Prionailurus_iriomotensis         . . . . . . . . . . . . . . . . . . . .
Prionailurus_viverrinus           . . . . . . . . . . . . . . . . . . . .
Acinonyx_jubatus                  . . . . . . . . . . . . . . . . . . . .
Puma_concolor                     . . . . . . . . . . . . . . . . . . . .
Puma_yagouaroundi                 . . . . . . . . . . . . . . . . . . . .
Lynx_canadensis                   . . . . . . . . . . . . . . . . . . . .
Lynx_pardinus                     . . . . . . . . . . . . . . . . . . . .
Lynx_rufus                        . . . . . . . . . . . . . . . . . . . .
Leopardus_geoffroyi               . . . . . . . . . T . . . . . . . . . .
Caracal_caracal                   . . . . . . . . . . . . . . . . . . . .
Neofelis_diardi                   . . . . . . . . . . . . . . . . . . . .
Neofelis_nebulosa                 . . . . . . . . . . . . . . . . . . . .
Panthera_leo                      . . . . . . . . . . . . . . . . . . . .
```

										330										340
Panthera_onca	.	.	.	.	.	.	.	.	.	.	.	.	.	.	.	.	.	.	.	.
Panthera_pardus	.	.	.	.	.	.	.	.	.	.	.	.	.	.	.	.	.	.	.	.
Panthera_pardus_nimr	.	.	.	.	.	.	.	.	.	.	.	.	.	.	.	.	.	.	.	.
Panthera_tigris_altaica	.	.	.	.	.	.	.	.	.	.	.	.	.	.	.	.	.	.	.	.
Panthera_tigris_jacksoni	.	.	.	.	.	.	.	.	.	.	.	.	.	.	.	.	.	.	.	.
Panthera_tigris_tigris	.	.	.	.	.	.	.	.	.	.	.	.	.	.	.	.	.	.	.	.
Panthera_uncia	.	.	.	.	.	.	.	.	.	T	.	.	.	.	.	.	.	.	.	.
Crocuta_crocuta	.	.	.	.	.	.	.	.	.	T	.	.	.	.	.	.	.	.	.	.
Hyaena_hyaena	.	.	.	.	.	.	.	.	.	T	.	.	.	.	.	.	.	.	.	.
Proteles_cristata_cristata	.	.	.	.	.	.	.	.	.	T	.	.	.	.	.	.	.	.	.	.
Canis_lupus_familiaris	.	.	.	.	.	.	.	.	.	T	.	.	.	.	.	.	.	.	.	.
Ailuropoda_melanoleuca	.	.	.	.	.	.	.	.	.	T	.	.	.	.	.	.	.	.	.	.
Paguma_larvata	.	.	.	.	.	.	.	.	.	T	.	.	C	.	.	C	.	.	.	.
Paradoxurus_hermaphroditus	.	.	.	.	.	.	.	.	.	T	.	.	.	.	.	C	.	.	.	.
Neovison_vison	.	.	.	.	.	.	.	.	.	T	.	.	G	.	.	C	.	.	.	.
Potos_flavus	.	.	.	T	.	.	.	.	.	T	.	.	.	.	.	.	.	.	.	.
Desmodus_rotundus	.	.	.	T	.	.	.	.	.	T	.	.	.	.	.	.	.	.	.	.
Sarcophilus_harrisii	.	.	.	.	.	.	.	.	.	.	.	.	C	.	.	C	.	.	G	.
Sminthopsis_crassicaudata	.	.	.	.	.	.	.	.	.	.	.	.	C	.	.	C	.	.	G	.
Phascolarctos_cinereus	.	.	.	.	.	.	.	.	.	T	.	.	C	.	.	C	.	.	G	.
Erinaceus_europaeus	.	.	.	G	.	.	.	.	.	T	.	.	A	.	.	.	.	.	.	.
Talpa_europaea	.	.	.	T	.	.	.	.	.	T	.	.	.	.	.	.	.	.	.	.
Ornithorhynchus_anatinus	.	.	.	.	.	.	.	.	.	T	.	.	C	.	.	C	.	.	G	.
Tachyglossus_aculeatus	.	.	.	.	.	.	.	.	.	T	.	.	C	.	.	C	.	.	G	.
Macaca_fascicularis	.	.	.	T	.	.	.	.	.	T	.	.	.	.	.	.	.	.	.	.
Homo_sapiens	.	.	.	T	.	.	.	.	.	T	.	.	.	.	.	C	.	.	.	.
Pan_troglodytes	.	.	.	T	.	.	.	.	.	T	.	.	.	.	.	C	.	.	.	.
Mus_musculus	.	.	.	.	.	.	.	.	.	T	.	.	A	.	.	.	.	.	.	.
Tupaia_belangeri	.	.	.	.	.	.	.	.	.	T	.	.	.	.	.	.	.	.	.	.
Chelonia_mydas	.	.	.	A	.	.	.	.	.	T	.	.	.	.	.	.	.	.	G	.
Oryzias_latipes	.	.	.	.	.	.	.	.	.	.	.	.	C	.	.	C	.	.	G	.
Carassius_auratus	.	.	.	.	.	.	.	.	.	T	.	.	C	.	.	C	.	.	G	.
Ambystoma_mexicanum	.	.	.	.	.	.	.	.	.	T	.	.	.	.	.	.	.	.	.	.
Anas_platyrhynchos	.	.	.	A	.	.	.	.	.	T	.	.	G	.	.	.	.	.	G	.
Cairina_moschata	.	.	.	A	.	.	.	.	.	T	.	.	A	.	.	.	.	.	G	.
Oceanodroma_leucorhoa	.	.	.	A	.	.	.	.	.	T	.	.	A	.	.	.	.	.	G	.
Pygoscelis_papua	.	.	.	A	.	.	.	.	.	T	.	.	A	.	.	.	.	.	G	.
Spheniscus_humboldti	.	.	.	A	.	.	.	.	.	T	.	.	A	.	.	.	.	.	G	.
Struthio_camelus	.	.	.	A	.	.	.	.	.	T	.	.	A	.	.	.	.	.	G	.
Scyliorhinus_canicula	.	.	.	T	.	.	.	.	.	T	.	.	A	.	.	C	.	.	.	.
Leucoraja_erinacea	.	.	.	T	.	.	.	.	.	.	.	.	C	.	.	C	.	.	.	.
Felis_catus	T	G	G	A	A	T	C	T	C	A	G	T	T	G	A	A	A	A	A	C
Felis_chaus	.	.	.	.	.	.	.	.	.	.	.	.	.	.	.	.	.	.	.	.

Felis_nigripes	....
Otocolobus_manul	....
Prionailurus_bengalensis	....
Prionailurus_bengalensis_eu	....
Prionailurus_iriomotensis	....
Prionailurus_viverrinus	....
Acinonyx_jubatus	....
Puma_concolor	....
Puma_yagouaroundi	....
Lynx_canadensis	....
Lynx_pardinus	....
Lynx_rufus	....
Leopardus_geoffroyi	....
Caracal_caracal	....
Neofelis_diardi	...................T
Neofelis_nebulosa	...................T
Panthera_leo	...................T
Panthera_onca	...................T
Panthera_pardus	...................T
Panthera_pardus_nimr	...................T
Panthera_tigris_altaica	...................T
Panthera_tigris_jacksoni	...................T
Panthera_tigris_tigris	...................T
Panthera_uncia	...................T
Crocuta_crocuta	...................T
Hyaena_hyaena	...................T
Proteles_cristata_cristata	...................T
Canis_lupus_familiaris	...................T
Ailuropoda_melanoleuca	...................T
Paguma_larvata	...................T
Paradoxurus_hermaphroditus	...................T
Neovison_vison	...................T
Potos_flavus	...................T
Desmodus_rotundus	...................T
Sarcophilus_harrisii	....G..C...C...G..
Sminthopsis_crassicaudata	....G..C...C...
Phascolarctos_cinereus	....G..A...C...
Erinaceus_europaeus	.A..G........A......
Talpa_europaea	...................T
Ornithorhynchus_anatinus	...........C.A....
Tachyglossus_aculeatus	....G......C.A....
Macaca_fascicularis	....G.............GT
Homo_sapiens	...................T
Pan_troglodytes	...................T
Mus_musculus	.A..G..C..AC......GT
Tupaia_belangeri	...................T

```
Chelonia_mydas                 . . . . . . . . . . A C . . . . . . C T
Oryzias_latipes                .
Carassius_auratus              . A . .
Ambystoma_mexicanum            . . . . G . . . . . . C . . . . . . C A
Anas_platyrhynchos             . . . . . . . G . . A C . . . . . . . T
Cairina_moschata               . . . . . . . G . . A C . . . . . . . T
Oceanodroma_leucorhoa          . . . . . . . A . . A C . . . . . . . T
Pygoscelis_papua               . . . . . . . A . . A C . . . . . . . T
Spheniscus_humboldti           . . . . . . . A . . A C . . . . . . . T
Struthio_camelus               . . . . . . . A . . A C . . . . . . . .
Scyliorhinus_canicula          . . . .
Leucoraja_erinacea             . . . .


                               . . . . |
Felis_catus                    A T T A G
Felis_chaus                    . . . . .
Felis_nigripes                 . . . . .
Otocolobus_manul               . . . . .
Prionailurus_bengalensis       . . . . .
Prionailurus_bengalensis_eu    . . . . .
Prionailurus_iriomotensis      . . . . .
Prionailurus_viverrinus        . . . . .
Acinonyx_jubatus               . . . . .
Puma_concolor                  . . . . .
Puma_yagouaroundi              . . . . .
Lynx_canadensis                . . . . .
Lynx_pardinus                  . . . . .
Lynx_rufus                     . . . . .
Leopardus_geoffroyi            . . . . .
Caracal_caracal                . . . . .
Neofelis_diardi                . . . . .
Neofelis_nebulosa              . . . . .
Panthera_leo                   . . . . .
Panthera_onca                  . . . . .
Panthera_pardus                . . . . .
Panthera_pardus_nimr           . . . . .
Panthera_tigris_altaica        . . . . .
Panthera_tigris_jacksoni       . . . . .
Panthera_tigris_tigris         . . . . .
Panthera_uncia                 . . . . .
Crocuta_crocuta                . . . . .
Hyaena_hyaena                  . . . . .
Proteles_cristata_cristata     . . . . .
Canis_lupus_familiaris         . . . . .
Ailuropoda_melanoleuca         . . . . .
```

Paradoxurus_hermaphroditus	. . . . .
Neovison_vison	. . . . .
Potos_flavus	. . . . .
Desmodus_rotundus	. . . . .
Sarcophilus_harrisii	
Sminthopsis_crassicaudata	
Phascolarctos_cinereus	
Erinaceus_europaeus	. . C . .
Talpa_europaea	. . . . .
Ornithorhynchus_anatinus	
Tachyglossus_aculeatus	
Macaca_fascicularis	. . . . .
Homo_sapiens	. . . . .
Pan_troglodytes	. . . . .
Mus_musculus	. . . . .
Tupaia_belangeri	. . . . .
Chelonia_mydas	. . . . .
Oryzias_latipes	
Carassius_auratus	
Ambystoma_mexicanum	. . . . .
Anas_platyrhynchos	. . . . .
Cairina_moschata	. . . . .
Oceanodroma_leucorhoa	. . . . .
Pygoscelis_papua	. . . . .
Spheniscus_humboldti	. . . . .
Struthio_camelus	. . . . .
Scyliorhinus_canicula	
Leucoraja_erinacea	

C

Figure S1: Annotated alignments of microsatellite loci.

(a) Ptia5: 5'-flanking region (1-204), repeat motif (204-205, details for Figure 1), 3'-flanking region (205-234).

(b) Ptia2-intron: 5'-flanking region (1-80), repeat motif (80-81, details for Figure 2), 3'-flanking region (81-124).

(c) Ptia2-exon: 5'-flanking region (1-30), repeat motif (31-91), 3'-flanking region (92-345).

Gap symbols: "-" = indel; "." = identity to reference sequence.
